# Designing a multi-epitope vaccine candidate against human rhinovirus C utilizing immunoinformatics approach

**DOI:** 10.3389/fimmu.2024.1364129

**Published:** 2025-01-07

**Authors:** Tajul Islam Mamun, Md. Ahad Ali, Md. Nazmul Hosen, Jillur Rahman, Md. Anwarul Islam, Md. Golam Akib, Kamruz Zaman, Md. Masudur Rahman, Ferdaus Mohd Altaf Hossain, Samir Ibenmoussa, Mohammed Bourhia, Turki M. Dawoud

**Affiliations:** ^1^ Department of Epidemiology and Public Health, Sylhet Agricultural University, Sylhet, Bangladesh; ^2^ Faculty of Veterinary, Animal and Biomedical Sciences, Sylhet Agricultural University, Sylhet, Bangladesh; ^3^ Department Of Chemistry, University of Rajshahi, Rajshahi, Bangladesh; ^4^ Department of Pharmacology and Toxicology, Sylhet Agricultural University, Sylhet, Bangladesh; ^5^ Department of Pathology, Sylhet Agricultural University, Sylhet, Bangladesh; ^6^ Department of Dairy Science, Sylhet Agricultural University, Sylhet, Bangladesh; ^7^ Laboratory of Therapeutic and Organic Chemistry, Faculty of Pharmacy, University of Montpellier, Montpellier, France; ^8^ Department of Chemistry and Biochemistry, Faculty of Medicine and Pharmacy, Ibn Zohr University, Laayoune, Morocco; ^9^ Department of Botany and Microbiology, College of Science, King Saud University, Riyadh, Saudi Arabia

**Keywords:** human rhinovirus C, multi-epitope vaccine, epitope prediction, reverse vaccinology, immunoinformatics

## Abstract

Human rhinovirus C (HRV-C) is a significant contributor to respiratory tract infections in children and is implicated in asthma exacerbations across all age groups. Despite its impact, there is currently no licensed vaccine available for HRV-C. Here, we present a novel approach to address this gap by employing immunoinformatics techniques for the design of a multi-epitope-based vaccine against HRV-C. The sequences of the chosen structural proteins VP1 and VP2, along with the non-structural protein 2C of HRV-C, were downloaded in FASTA format from the NCBI server for further analysis. Through an exhaustive analysis of HRV-C genomic sequences, we identified highly conserved immunogenic regions capable of eliciting a protective immune response. Leveraging advanced immunoinformatics tools, we predicted epitopes for B-cells, Cytotoxic T lymphocytes, and Helper T lymphocytes, ensuring broad coverage across different HRV-C strains. The vaccine candidate was constructed by integrating selected antigens with immunogenic epitopes and adjuvants, employing optimal linkers. Three vaccine constructs were developed, with V2 being the most promising, consisting of 480 amino acids residues. V2 exhibited strong antigenicity, non-allergenicity, and solubility, with a solubility score greater than 0.550, and demonstrated excellent structural stability, with 91.9% of residues in the most favorable regions of the Ramachandran plot. Molecular dynamics and simulation studies revealed a stable Vaccine-TLR8 complex, with a binding energy of -296.15 and consistent RMSD values. Furthermore, in silico cloning and sequence optimization ensured efficient expression in *E. coli*, with a Codon Adaptation Index of 0.99 and GC content of 54.58%. The minimum free energy of the RNA secondary structure was -494.90 kcal/mol. While our findings suggest the potential effectiveness of the designed vaccine candidate against HRV-C, further *in vitro* and *in vivo* investigations are warranted to validate its safety and efficacy.

## 1 Introduction

Human rhinoviruses (HRVs), the causative agent of the common cold, are highly prevalent respiratory viruses that are responsible for respiratory tract infections and exacerbations of chronic respiratory disorders, including chronic obstructive pulmonary disease (COPD) and asthma (1). They account for over 50% of common cold cases, leading to significant annual economic losses exceeding $60 billion (2, 3). This virus belongs to the picornaviridae family and is a positive-sense ssRNA virus that is non-enveloped (4). To date, 171 genotypes of rhinovirus have been recognized and categorized into three genetic groups (A, B, or C) based on sequence similarity (5–7). Although HRV A and C genotypes exhibit equivalent incidence and infection rates, the most recent study has shown that HRV C plays a significant role in developing asthma and COPD, in contrast to HRV A (8, 9). According to the WHO, the prevalence of asthma in 2019 was estimated to be around 260 million individuals, resulting in 461,000 fatalities (10). The discovery of RV-C in 2006 using molecular techniques surprised scientists, as it had previously evaded detection by traditional methods. HRV-C variants are particularly significant due to their association with childhood pneumonia, wheezing, and the common cold (11, 12).

The viral proteome consists of 11 proteins, including the structural proteins VP1, VP2, and VP3 that form the capsid, while non-structural proteins aid in genome replication (13). The virus’s antigenic diversity primarily arises from the external structural proteins VP1, VP2, and VP3, while VP4, found inside the capsid, exhibits minimal variation (14). Recent advancements in genome sequencing and bioinformatics have enabled the application of immunoinformatics and reverse vaccinology to vaccine design (15, 16). Current medications do not adequately serve the treatment and prevention of these acute occurrences of viral infections (17). Vaccination is the most effective strategy to combat the transmission and effects of novel viral pathogens, particularly HRV, which leads to significant economic costs and unmet medical needs. Despite the clinical importance of HRV-C, developing effective vaccines is challenging due to genetic diversity, host-virus interactions, viral mutations, and a lack of clear correlation with protective immunity. Recent progress in genome sequencing and bioinformatics has made it possible for researchers to combine immunoinformatics and reverse vaccinology to solve problems in vaccine design (18).

Computational methods have been employed to design vaccines for various viruses, including Dengue, Ebola, Oropouche, Hepatitis C, human coronaviruses, Lassa, and Saint Louis encephalitis (19–22). This strategy integrates the fields of immunogenomics and immunoinformatics to identify undiscovered targets of vaccine (23). We employed immunoinformatics techniques to develop a multi-epitope vaccine against HRV-C, offering a promising solution to the challenges faced in traditional vaccine development. By strategically selecting and combining diverse epitopes from various viral proteins, this multi-epitope vaccine aims to elicit a broader and more robust immune response. This approach leverages conserved epitopes to address the genetic diversity of HRV-C strains effectively. Moreover, integrating immunoinformatics into epitope prediction enhances the accuracy and efficacy of the vaccine design process.

This study employed various immunoinformatics tools to design and evaluate a multi-epitope vaccine against HRV-C. Protein sequences were sourced from NCBI, and antigenic properties were analyzed with VaxiJen. The IEDB server facilitated epitopes prediction for B-cells, CTL, and HTL, while Hdock assessed epitope-receptor interactions through molecular docking studies. Vaccine design incorporated epitopes with adjuvants using linker prediction tools, and GROMACS was used for molecular dynamics simulations to evaluate stability. The JCat server enabled in silico cloning and codon optimization for *E. coli* expression. This comprehensive approach not only enhances immune responses through the strategic combination of diverse viral epitopes but also reduces the risk of escape mutants, ultimately providing a flexible strategy to combat HRV-C effectively.

## 2 Methods and materials

**Figure 1:**
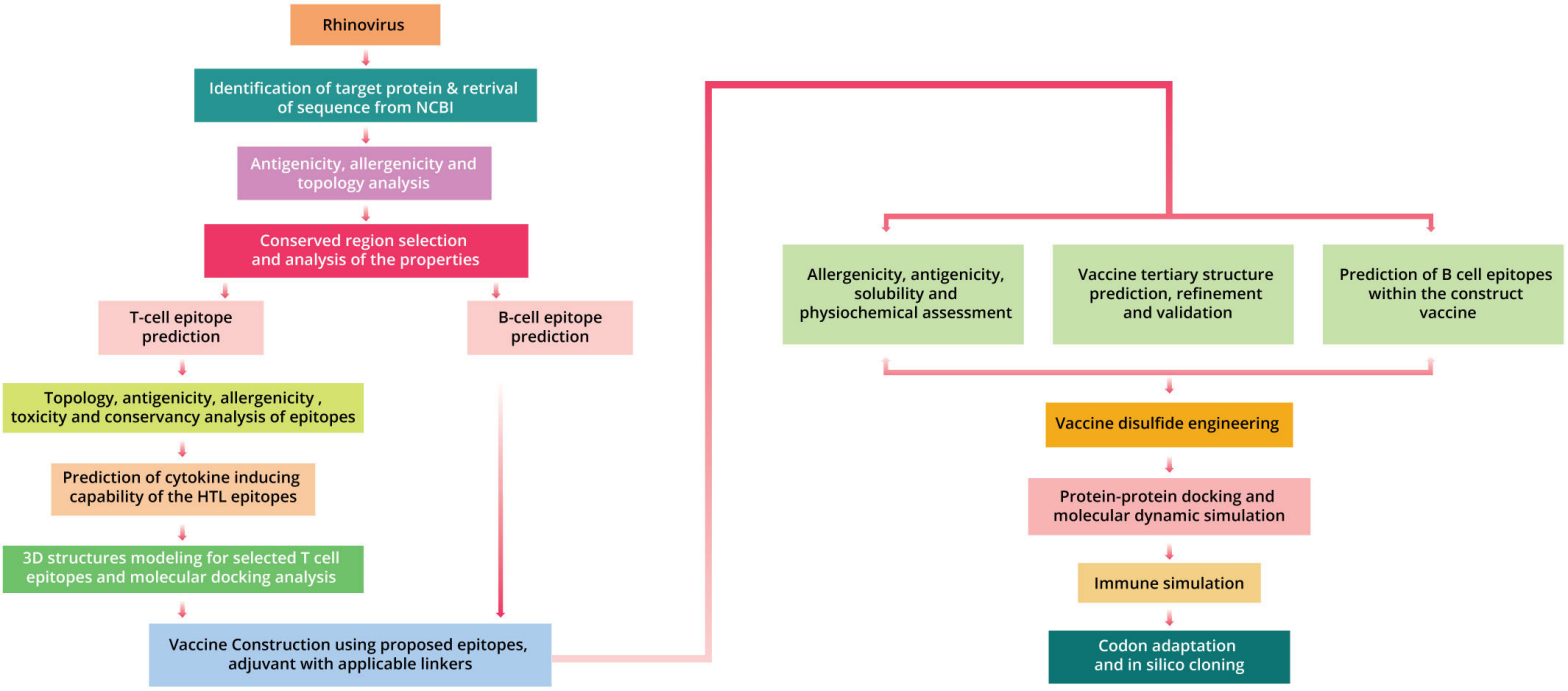
The complete procedure utilized for formulating a vaccine against human rhinovirus C.

Human rhinovirus (HRV-C) vaccine was designed by applying various computational methodologies in the research phase, based on numerous epitopes (**Supplementary Table 1**; **Figure 1**).

### 2.1 Acquiring protein sequences and recognizing the conserved region

Three proteins were chosen for vaccine design based on their allergenicity, antigenicity, and transmembrane structure after a thorough literature review. The sequences of the selected structural proteins HRV-C (VP1 and VP2) and the non-structural protein (2C) were retrieved in FASTA format from the NCBI server for subsequent analysis. Using NCBI BLAST, we identified identical sequences for the HRV-C strain (24). The identification of conserved regions among the homologous sequences was then conducted using the Clustal Omega server. Subsequently, antigenicity, allergenicity, and conserved region topological features were evaluated with the assistance of VaxiJen v2.0, AllerTop, and the TMHMM servers (25–27).

### 2.2 Determination of T-cell epitopes

T-cell epitopes were forecasted through the IEDB server, employing conserved regions (28). The length of the epitopes was set to 9 amino acids prior to the prediction step for Cytotoxic T Lymphocytes (CTL). This is a standard length for CTL epitopes, allowing for optimal binding and recognition by Major Histocompatibility Complex (MHC) class I molecules. After completing the CTL epitope prediction, the peptide length was set to 15 amino acids for Helper T Lymphocytes (HTL) prediction. This longer length is appropriate for HTL epitopes, which interact with MHC class II molecules and play a crucial role in the immune response by providing help to other immune cells, including B cells and CTLs. Epitopes were generated for CTL and HTL using MHC class I (HLA A, B, and C) and class II (HLA DR, DP, and DQ) alleles.

### 2.3 Determination of B-cell epitopes

IEDB’s B-cell epitope prediction server was applied to determine B-cell epitopes using a variety of methods, including the Karplus and Schulz flexibility scale. This approach helps identify regions of the protein that are likely to be accessible and flexible, which are key characteristics of effective B-cell epitopes (29, 30). Additionally, the ABCpred server was used to identify B-cell epitopes within the target proteins, employing default parameters (31).

### 2.4 Assessment of anticipated B-cell and T-cell epitopes

The primary selections were chosen based on their antigenicity, allergenicity, topological, and toxicity profiles among all epitopes identified by VaxiJen v2.0, AllerTOP, TMHMM, and ToxinPred servers (25–27, 32, 33). These criteria ensure the prioritization of epitopes with favorable characteristics for further vaccine design. Antigenicity was evaluated using VaxiJen v2.0 with a threshold of 0.4. Allergenicity was assessed using AllerTOP with the default threshold to exclude potential allergens. Topological features were determined using TMHMM to ensure epitopes are appropriately exposed on the viral surface. Toxicity was screened using ToxinPred to eliminate toxic epitopes, ensuring non-toxicity. For epitope lengths, CTL epitopes were set to 9 amino acids, HTL epitopes to 15 amino acids, and B-cell epitopes were evaluated with various methods to ensure flexibility and surface accessibility, generally ranging between 10-20 amino acids. These parameters ensure that the selected epitopes possess desirable characteristics for vaccine development, including immunogenicity, safety, and structural suitability.

### 2.5 Prediction of epitopes capable of inducing IFN-γ

Activation of natural killer cells and macrophages by antigen-induced IFN-γ is necessary for both innate and adaptive immunity (34). SVM method was employed to predict IFN-inducing epitopes, categorizing them as positive or negative inducers through the use of the IFN-epitope and IL4pred servers (35).

### 2.6 Analysis of epitope population coverage and conservation

In order to safeguard a wide-ranging population, it is crucial for vaccination initiatives to prioritize coverage across diverse communities and regions, taking into account the considerable variability in HLA profiles. The IEDB server was employed to conduct epitope population coverage and conservancy analysis. Within the epitope conservancy analysis, which concentrates on protein identities, the measurement of conservancy intensity was employed to identify optimal epitope locations within homologous protein groups. This comprehensive approach ensures a coherent strategy in assessing and addressing the diverse factors influencing vaccination effectiveness.

### 2.7 Docking for the chosen epitopes

The PEP-FOLD server was employed to predict the 3D structures of epitopes (36). Docking studies with MHC class-I and class-II epitopes were conducted for HLA A02:03 and HLA-DQ2.3, respectively, based on epitope-allele interaction data. The protein was initially purified or processed by applying Discovery Studio tool. Docking analyses for the binding affinity between predicted epitopes were performed using Autodock Vina software, where high binding energy epitopes were considered the best (37). For 3D visualization of molecular structures, we used UCSF Chimera software to gain detailed insights into the structural orientation and binding potential of our selected epitopes.

### 2.8 Multi-epitope vaccine development

The final vaccine candidate was designed by combining all the epitopes predicted by different immunoinformatic methods and adding the right adjuvants and linkers. Three possible vaccine candidates (V1, V2, and V3) were made by combining different adjuvants with PADRE sequences and the top HTL, CTL, and BCL epitopes. Adjuvants enhance immune responses and induce persistent reactions through diverse immunological pathways (38). Various adjuvants were employed, including β-adjuvant (Accession no. AF295370), HABA protein (Accession no. AGV15514.1), and 50S ribosomal protein L7/L12 (Accession no. P9WHE3). To ensure uniformity across all domains, epitopes, and adjuvants were interconnected using EAAAK, GGGS, GPGPG, and KK linkers.

### 2.9 Evaluation of the developed vaccine structure

The allergenicity of the developed vaccines was assessed using specific server, namely AllerTOP v2.0 (25). VaxiJen v2.0, ToxinPred and Protein-Sol servers were used to evaluate the vaccine’s antigenicity and solubility, respectively, to identify the best candidate (26, 33, 39). Additionally, the ExPASy website was utilized to determine the physical and chemical features of the vaccine candidate (40). This multi-faceted approach ensured a thorough evaluation of the vaccine candidates, enhancing the likelihood of selecting an effective and safe vaccine for further development. To evaluate the potential for long-term immunity, we utilized immunoinformatics tools such as IEDB’s Class I and II MHC Binding Prediction and MEMORY to predict memory B-cell and T-cell epitopes. These tools accelerated to assess the ability of the selected epitopes to induce durable immune responses, which are critical for sustained protection against HRV-C.

### 2.10 Analysis of secondary and tertiary structures prediction, along with the quality assessment of the constructed vaccine

The PSIPRED server, a highly accurate method for secondary structure prediction, was utilized to predict the alpha, beta-sheet, and coil structures of the vaccine construct (41). Its precision is rigorously assessed through cross-validation to measure effectiveness (42). Additionally, I-TASSER algorithms were employed to predict the vaccine’s tertiary structures (43). For refining the anticipated 3D structure, the GalaxyRefine server was utilized (44). The quality of the refined vaccine structure was further evaluated using a Ramachandran plot generated by the PROCHECK web server (45). Furthermore, the VERIFY 3D and ERRAT servers were employed to assess the predicted 3D structure (46, 47).

### 2.11 Implementation of disulfide engineering and forecasting of B-cell epitopes

Disulfide engineering involves introducing new disulfide bonds in proteins by modifying cysteine residues, thereby enhancing stability (48). To enhance vaccine stability, the DbD2 tool was employed to create disulfide bonds between potential residue pairs within the vaccine structure (49). Furthermore, the IEDB server’s ElliPro program, using default settings, was utilized to confirm the inclusion of linear and conformational B-cell epitopes in the vaccine design (50).

### 2.12 Molecular docking and simulation

The HDOCK server was employed to conduct comprehensive molecular docking studies aimed at evaluating the binding affinity and interaction dynamics between the newly designed multi-epitope vaccine and Toll-like receptor 4 (TLR4, PDB ID: 4G8A) as well as Toll-like receptor 8 (TLR8, PDB ID: 3W3M) (51). The GROMACS version 2022.3 performed a molecular dynamics simulation lasting 100 nanoseconds (ns). The simulation incorporated the CHARMM36m force field, and a water box was generated using the TIP3 water model, ensuring its edges were set at a distance of 1 nm from the protein surface. Neutralization of the systems was achieved by introducing the required ions. After the simulation, GROMACS software’s integrated modules (RMSD, RMSF, gyrate, SASA, and Hbond) were employed for analyzing root mean square deviation (RMSD), root mean square fluctuation (RMSF), radius of gyration (Rg), solvent accessible surface area (SASA), and hydrogen bonds. The analyses were visualized through graphs created using the ggplot2 package in RStudio (52, 53).

### 2.13 *In silico* cloning and codon adaptation

Codon adaptation using the JCAT website facilitated protein synthesis in *E. coli* K12 (54). Restriction sites for Eco53KI and HpaI were introduced at the N and C termini of the vaccine sequence, respectively. The SnapGene tool was used to compare the changed nucleotide sequence of the vaccine construct with the *E. coli* pET28a(+) expression vector as part of the cloning modeling process (55). Additionally, the RNAfold online tool was used to estimate thermodynamically low-free-energy mRNA secondary structures (56).

### 2.14 Immunological responses triggered by the vaccine candidates

The vaccine’s immunogenic properties were analyzed in a real-world situation using the C-ImmSim program (57). It’s an antigen-based model that predicts immunological responses using an artificial algorithm and position-specific score matrix. The typical guideline for most immunizations is to wait four weeks between doses (58). Three injections, each containing 1000 antigens, were provided at intervals of 8 weeks and 24 weeks following the first injection. These injections were given at 1, 252, and 504 time-steps, representing 8-hour real-life intervals. Other parameters remained the same, but simulation steps were increased to 1050.

## 3 Results

### 3.1 Protein sequence retrieval and analysis of conserved areas

Human rhinovirus (HRV-C) protein sequences were retrieved from the NCBI database in FASTA format. Three proteins, namely VP1, VP2, and 2C, were selected as potential vaccine targets for this study. Using the VaxiJen server with a cutoff of 0.4, the antigenic properties of these sequences were analyzed. The transmembrane structures of the selected proteins were found to be external, with no observed allergic responses. Physiochemical properties of these proteins are detailed in **Table 1**. To identify homologous proteins, BLASTp algorithms from NCBI were employed, generating multiple lists for each protein evaluated. Conserved regions within these proteins were scrutinized to facilitate the design of subsequent vaccines. Specifically, conserved regions with lengths exceeding 15 nucleotides were prioritized at this stage to expedite the development of a highly effective vaccine capable of targeting various HRV-C variants. The study focused on evaluating 15 specific conserved regions selected for their potential as antigens, allergens, and their transmembrane topology (**Supplementary Table 2**).

**Table 1:** Analysis of selected proteins from HRV-C.

Rhinovirus C Protein	Antigen score	No. of Amino acids	Molecular Weight	Theoretical pI	Aliphatic index	Grand average of hydropathicity	Instability Index	Accession
VP1	0.5871	273	30981.90	5.84	68.24	-0.493	35.59	AEM44647.1
VP2	0.5484	261	28777.52	6.34	85.86	-0.260	32.87	YP_001552433.1
2C	0.4219	326	36467.54	8.96	94.51	-0.279	39.34	YP_001552438.1

### 3.2 Effective T-cell epitope identification

T-cell epitopes are primarily composed of short peptides with the capacity to trigger an immune response. Cytotoxic T-cells (CTL) employ their specialized T-cell receptor to recognize specific antigens (59). A complex is formed when the antigen-specific T-cell receptor binds to the CTL epitope on the surface of virus-infected cells (60). The IEDB server employed identified conserved sequences to create T-cell epitopes for MHC class-I and MHC class-II across three proteins. Due to their high binding sensitivity and compatibility with a broad variety of HLAs, these epitopes were chosen for further study (**Supplementary Tables 3**–**5**). All CTL epitopes exhibit high antigenicity, non-allergenic, non-toxic, located externally, and show 100% conservancy, making them effective targets across different HRV-C strains. Seven CTL epitopes, each consisting of 9 amino acids, were selected as potential vaccine targets due to their high antigenicity and conserved nature (**Table 2**). A foreign antigen may trigger cellular immunity when the HLA class I molecule attaches to and delivers the peptides to CD8^+^ T-cells. The adaptive immune response is mediated mainly by helper T-cells. The HLA class II protein attaches to peptides that may generate humoral and cellular immunity against the pathogen and then delivers these peptides to CD4^+^ cells (61). Similarly, the HTL epitopes demonstrate high antigenicity, non-allergenic, non-toxic, and externally located, with 100% conservancy. These epitopes have been evaluated for their cytokine-inducing properties, indicating their potential to induce immune responses. Five epitopes were selected from a pool of anticipated helper T-cell epitopes, each consisting of 15 amino acids (**Table 3**). These findings highlight the strong potential of these epitopes in eliciting robust immune responses, making them promising candidates for developing a multi-epitope vaccine against HRV-C.

**Table 2:** Selected CTL epitopes from VP1, VP2 and 2C.

Protein Name	Epitope sequence	Antigenicity	Allergenicity	Toxicity	Topology	Conservancy	HLA class I binding alleles
VP1	ALHDKDPRL	1.3654	Non-allergen	Non-toxic	Outside	100%	HLA-A*02:03
	GDFNLLGIH	1.3551	Non-allergen	Non-toxic	Outside	100%	HLA-B*40:01
	PAALHDKDP	1.4635	Non-allergen	Non-toxic	Outside	100%	HLA-C*08:02
VP2	ALEDKGKSF	1.0795	Non-allergen	Non-toxic	Outside	100%	HLA-C*02:02
	DLNTSAGYP	0.7451	Non-allergen	Non-toxic	Outside	100%	HLA-A*68:02
2C	LNTSAGYPY	0.6086	Non-allergen	Non-toxic	Outside	100%	HLA-A*30:02
	AGGENHVAF	0.4879	Non-allergen	Non-toxic	Outside	100%	HLA-C*03:02

**Table 3:** Selected HTL epitopes with their cytokine inducing properties.

Protein Name	Epitope sequence	Antigenicity	Allergenicity	Toxicity	Topology	Conservancy	INFpred	IL4pred
VP1	DLNTSAGYPYVTLGI	1.0886	Non-allergen	Non-toxic	Outside	100%	-0.621	IL4inducer
	LNTSAGYPYVTLGIK	1.3439	Non-allergen	Non-toxic	Outside	100%	-0.789	IL4inducer
VP2	IHDNCAVLPTHAECG	0.5347	Non-allergen	Non-toxic	Outside	100%	-0.613	IL4inducer
	GIHDNCAVLPTHAEC	0.4831	Non-allergen	Non-toxic	Outside	100%	-0.432	IL4inducer
2C	LCLLAWHNGKQQYED	0.6081	Non-allergen	Non-toxic	Outside	100%	-0.579	IL4inducer

### 3.3 Effective B cell epitope identification

B-cell epitopes are the primary focus of vaccine designed to prevent any given disease. B-cell epitopes must be antigenic and surface-accessible to elicit a robust immune response. The selected BCL epitopes from VP1, VP2, and 2C were identified based on their antigenicity, allergenicity, and toxicity profiles. For VP1, the epitopes are KEPAALHDKD with an antigenicity of 1.2349. All selected epitopes are non-allergenic and non-toxic, making them promising candidates for vaccine design (**Table 4**). The similar epitopes for the B-cell receptor release humoral or cellular immunity antibodies, making them pivotal in vaccine development and production (62, 63).

**Table 4:** Selected BCL epitopes from VP1, VP2 and 2C.

Protein Name	Epitope sequence	Antigenicity	Allergenicity	Toxicity
VP1	KEPAALHDKD	1.2349	Non-allergen	Non-toxic
	YPYVTL	1.0891	Non-allergen	Non-toxic
	EPAALHDKDPRLDLNT	0.8258	Non-allergen	Non-toxic
VP2	QDLNTSAGYPYVTLGI	1.0594	Non-allergen	Non-toxic
2C	KSLCLLAWHNGKQQYE	0.7052	Non-allergen	Non-toxic

### 3.4 Assessment of all epitopes for vaccine design

The vaccine was designed using multiple epitope subunits, rigorously tested against specific criteria for Helper T-cell (HTL), Cytotoxic T Lymphocyte (CTL), and B-cell epitopes. The best epitopes, selected based on antigenicity scores, allergenicity, toxicity, and transmembrane topology, were used to create a vaccine against Rhinovirus type C (**Tables 2**, **3**). Predicting HTL epitopes are crucial for developing both preventive and therapeutic vaccines. HTL epitopes stimulate cytokine production, which regulates nearly all adaptive immune responses. Five HTL epitopes were selected for their ability to stimulate IFN-γ, essential for the immune response by activating B-cells to produce antibodies, macrophages to eliminate pathogens, and CTLs to target infected cells. These epitopes were further evaluated for their capacity to induce cytokines, IFN-γ and IL-4, using the IFNepitope and IL4pred servers (**Table 3**). IFN stimulates Th-1 responses, leading to apoptosis induction, antigen-presenting cell activation, and upregulation of HLA class I and II molecules, while IL-4 stimulates Th-2 responses (64). The chosen epitopes satisfy all the specified criteria.

### 3.5 Epitopes conservancy and population coverage analyses

The conservation level of an epitope is a crucial factor in its capacity to generate wide-ranging protection. This investigation aimed to assess the extent of changes in the most probable epitopes over time, highlighting the importance of high conservation for vaccine design (32). The proteins contained numerous highly conserved epitopes, with some exhibiting a 100% conservation level (**Tables 2**, **3**). Population coverage analysis revealed that South Africa had the least significant coverage at 34.43%, while West Africa had the most significant coverage at 92.56% (**Supplementary Table 7**). The predicted epitopes covered a significant 68.6% of the global population, indicating a potential for substantial global impact.

### 3.6 T-cell epitopes underwent molecular docking studies for interaction analysis

**Figure 2:**
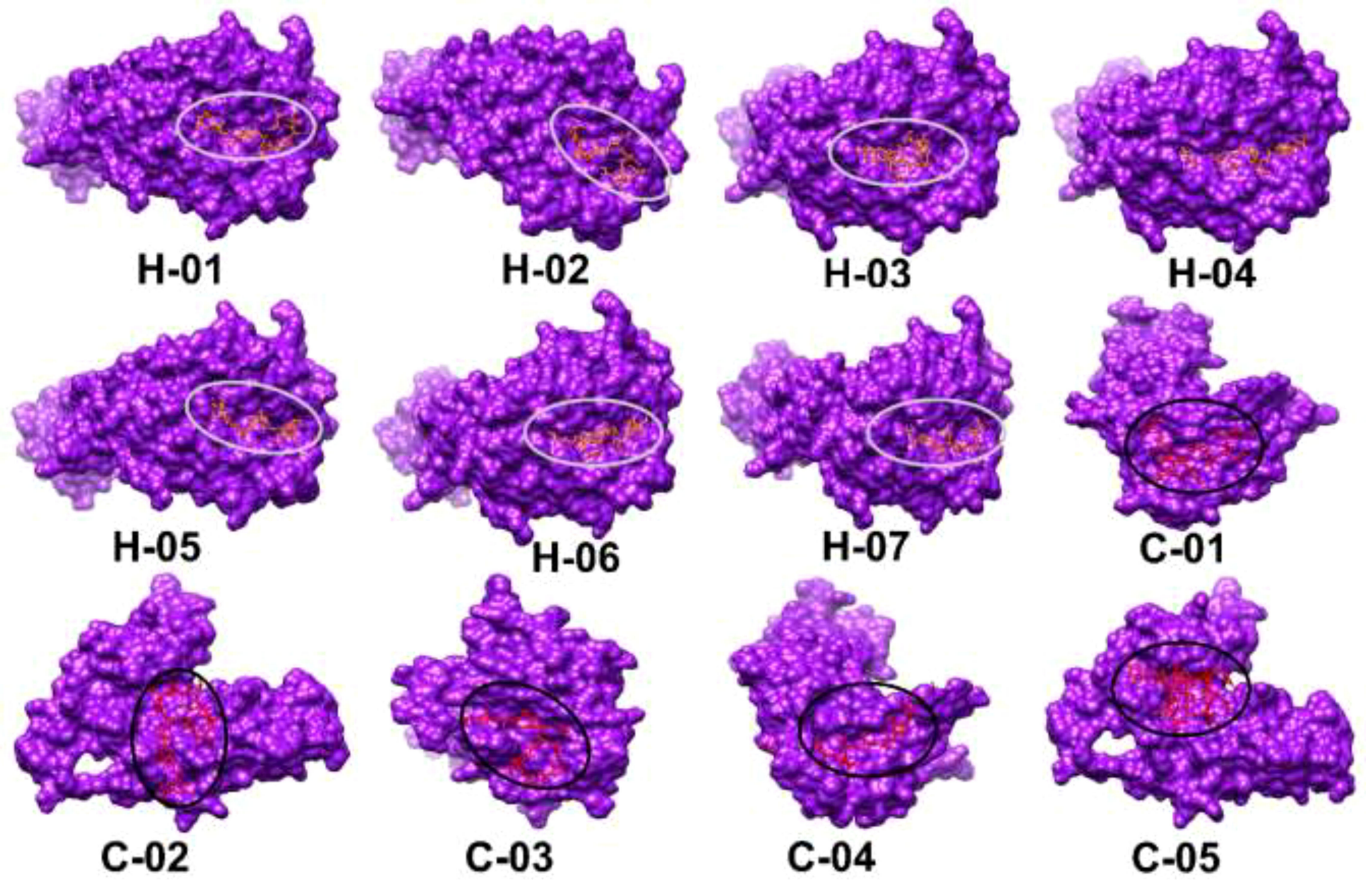
Three-dimensional structural models of selected epitopes (H-01 to H-07, C-01 to H-05) visualized using UCSF Chimera software. Each epitope is shown as a purple surface with binding sites highlighted in red and circled. Molecular docking results display HTL epitopes with HLA-DQ2.3 and CTL epitopes with HLA A*02:03.

Molecular docking plays a crucial role in evaluating the interaction and binding affinity between receptor and ligand molecules. Ensuring broad population coverage in vaccine design requires epitopes that exhibit high binding affinity for diverse alleles. In this study, we focused on HLA A*02:03 for molecular docking, identifying CTL epitopes that showed experimentally confirmed binding interactions with a majority of the selected epitopes (**Figure 2**). Docking studies assessed the binding performance of seven selected CTL epitopes, highlighting their top binding interactions (**Table 5**). Subsequently, we selected HLA-DQ2.3 for HTL epitopes, which also demonstrated experimentally verified binding interactions with most of the chosen epitopes. The binding performance of the selected HTL epitopes was deemed satisfactory (**Table 6**).

**Table 5:** Molecular docking result of HTL epitopes with HLA-DQ2.3(PDB: 4D8P).

SL No.	HTL binding peptide	Docking score (Kcal/mol)	RMSD (Å)	NHBs	Interacting residuals
1	DLNTSAGYPYVTLGI	-11.9	0.00	17	ASN11; TYR13; LEU70; GLY9; ALA7; ASN11; ASN111; ASP142; LEU66; SER8; LEU12; SER144; and ALA82; PRO115; PHE113; ILE74; ALA81; LEU66; LEU70
2	LNTSAGYPYVTLGIK	-11.2	0.00	11	TYR16; HIS167; TRP168; VAL117; ASN118; ASP171; ASP35; LYS147; LYS147; GLU166; ILE119; TRP168; LEU36
3	IHDNCAVLPTHAECG	-7.2	0.00	17	SER8; ASN11; SER15; THR83; SER139; SER141; HIS143; ASN84; VAL10; TYR13; PHE113
4	GIHDNCAVLPTHAEC	-12.9	0.00	15	TYR13; GLN14; TYR16; ALA82; SER141; HIS143; SER79; VAL73; GLU25; SER139; ALA3; SER141; LYS140; and ALA82; PRO114; TRP168
5	LCLLAWHNGKQQYED	-12.5	0.00	20	TYR33; ARG38; GLU37; ASN118; GLN14; GLN21; GLY20; VAL34; GLU134; SER19; PRO18; ILE63; LEU36

**Table 6:** Molecular docking result of CTL epitopes with HLA A*02:03 (PDB:3OX8).

SL No.	CTL binding peptide	Docking score (Kcal/mol)	RMSD (Å)	NHBs	Interacting residuals
1	ALHDKDPRL	-7.7	0.00	12	TYR7; LYS66; GLU63; ALA158; ASP77; VAL76; THR73; HIS70; LEU81; PHE9; TYR84; TYR123; TRP147; TRP156; THR80
2	GDFNLLGIH	-9.2	0.00	11	HIS70; TYR123; LYS146; ASP77; VAL76; HIS70; LYS66; TRP147; ALA69
3	PAALHDKDP	-10.6	0.00	17	TYR7; LYS66; HIS70; THR163; TYR99; GLN155; ARG97; TYR159
4	ALEDKGKSF	-10.8	0.00	18	TYR7; ARG97; ARG97; TRP147; TRP156; TYR171; ASP77; GLU63; TYR59; LU63; LYS66; TRP167; TRP167; LEU81
5	DLNTSAGYP	-9.5	0.00	15	THR73; THR80; LYS146; TRP147; TRP156; TYR171; TYR59; ASP77; TYR116; ASP77; GLU152; HIS70; THR143; LYS66; TRP156; TRP167; VAL76;
6	LNTSAGYPY	-9.7	0.00	10	TYR7; HIS70; THR73; TYR99; LYS146; LYS146; TRP147; GLU63; LYS66; GLU63; TRP147; TYR159; TRP156; LEU81; ATYR116; TYR123
7	AGGENHVAF	-9.6	0.00	15	LYS66; TYR99; TRP147; THR163; ASP77; GLU63; HIS70; LYS146; TRP167; TRP147; TRP147; TRP147; TRP167; TRP147

### 3.7 Designing vaccine based on epitopes

**Figure 3:**
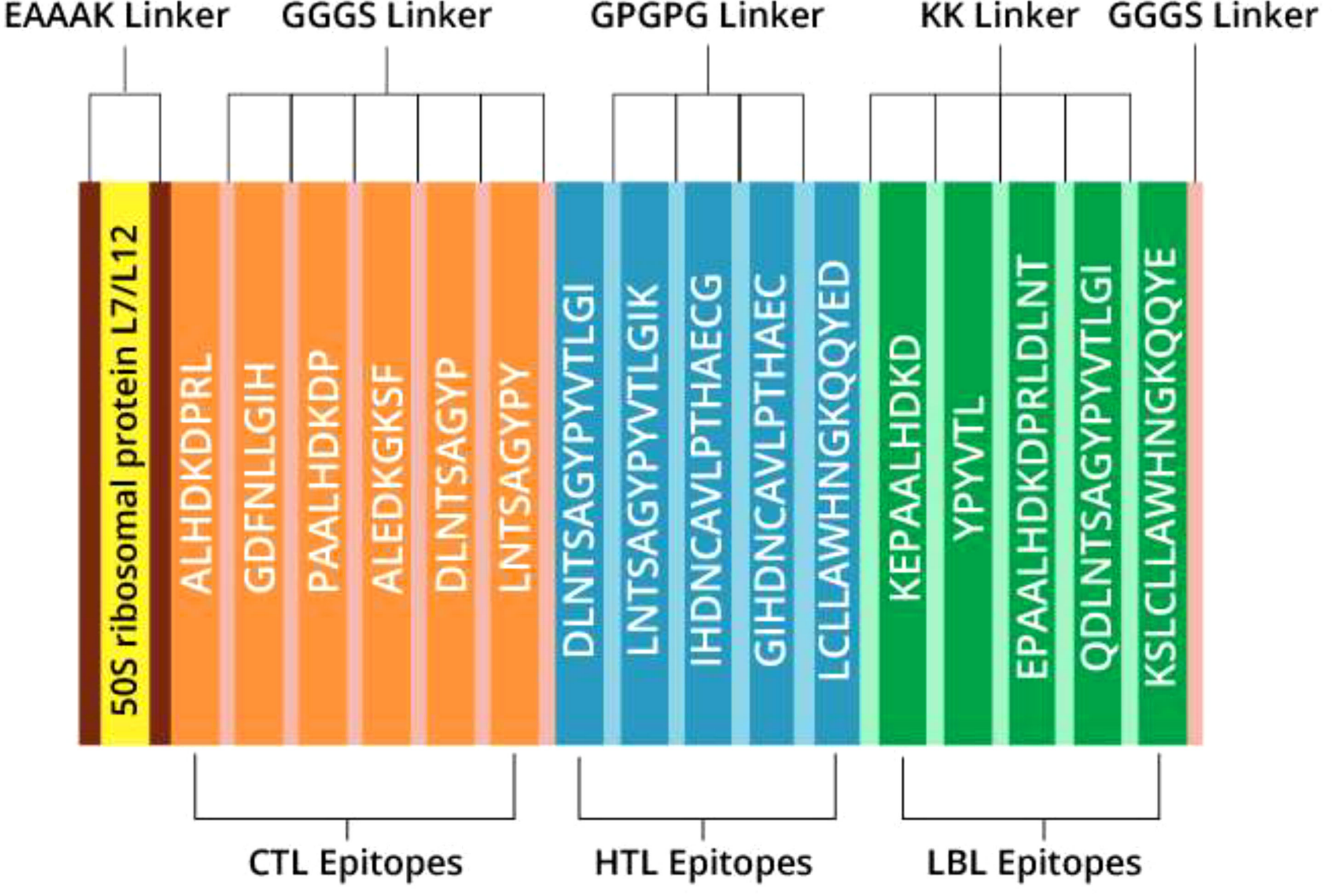
Illustration outlining the construction of the multi-epitope vaccine (V1).

The most promising epitopes and adjuvants were selected to construct the vaccine candidate. Each vaccine contains 7 CTL epitopes, 5 HTL epitopes, and 5 B-cell epitopes linked together using GGGS, GPGPG, and KK linkers, respectively. To enhance immunogenicity, the 50S ribosomal protein L7/L12 was attached to the N-terminus of the vaccine structure via the EAAAK linker (**Figure 3**). Vaccine candidates V2 and V3 were designed using the HABA protein and β-defensin-3 adjuvant, respectively (**Table 7**). The lengths of vaccine candidates V1, V2, and V3 are 405, 480, and 320 residues, respectively. The prediction of memory B-cell and T-cell epitopes suggests that our multi-epitope vaccine candidate has the potential to elicit not only immediate but also long-lasting immune responses.

**Table 7:** Evaluation of constructed vaccines.

Vaccine Construct	Composition	Complete Sequence of Vaccine Constructs	Allergenicity	VaxiJen score	Solubility
V1	Selected epitopes and 50S ribosomal protein L7/L12 adjuvant	**EAAAK**MAKLSTDELLDAFKEMTLLE LSDFVKKFEETFEVTAAAPVAVAAAG AAPAGAAVEAAEEQSEFDVILEAAGD KKIGVIKVVREIVSGLGLKEAKDLVD GAPKPLLEKVAKEAADEAKAKLEAA GATVTVK**EAAAK**ALHDKDPRLGGGS GDFNLLGIHGGGSPAALHDKDPGGGS ALEDKGKSFGGGSDLNTSAGYPGGGS LNTSAGYPYGGGSAGGENHVAF**GPGP** **G**DLNTSAGYPYVTLGI**GPGPG**LNTSA GYPYVTLGIK**GPGPG**IHDNCAVLPTHA ECG**GPGPG**GIHDNCAVLPTHAEC**GPG** **PG**LCLLAWHNGKQQYED**KK**KEPAAL HDKD**KK**YPYVTL**KK**EPAALHDKDPR LDLNT**KK**QDLNTSAGYPYVTLGI**KK**K SLCLLAWHNGKQQYE**GGGS**	Non allergen	0.6317	0.578
V2	Selected epitopes and heparin-binding hemagglutininadjuvant	**EAAAK**MAENPNIDDLPAPLLAALGAA DLALATVNDLIANLRERAEETRAETRT RVEERRARLTKFQEDLPEQFIELRDKFT TEELRKAAEGYLEAATNRYNELVERGE AALQRLRSQTAFEDASARAEGYVDQA VELTQEALGTVASQTRAVGERAAKLV GIELPGKAEAAGKKAQKAIAKAPAKK ASAKKAPAKKAPAKKAAAKKVTQK**E** **AAAK**ALHDKDPRLGGGSGDFNLLGIH GGGSPAALHDKDPGGGSALEDKGKSF GGGSDLNTSAGYPGGGSLNTSAGYPY GGGSAGGENHVAF**GPGPG**DLNTSAG YPYVTLGI**GPGPG**LNTSAGYPYVTLG IK**GPGPG**IHDNCAVLPTHAECG**GPGP** **G**GIHDNCAVLPTHAEC**GPGPG**LCLLA WHNGKQQYED**KK**KEPAALHDKD**KK** YPYVTL**KK**EPAALHDKDPRLDLNT**KK** QDLNTSAGYPYVTLGI**KK**KSLCLLAW HNGKQQYE**GGGS**	Non allergen	0.5713	0.556
V3	Selected epitopes and beta-defensin 3 adjuvant	**EAAAK**GIINTLQKYYCRVRGGRCAVL SCLPKEEQIGKCSTRGRKCCRRKK**EA** **AAK**ALHDKDPRLGGGSGDFNLLGIH GGGSPAALHDKDPGGGSALEDKGKS FGGGSDLNTSAGYPGGGSLNTSAGY PYGGGSAGGENHVAF**GPGPG**DLNTS AGYPYVTLGI**GPGPG**LNTSAGYPYV TLGIK**GPGPG**IHDNCAVLPTHAECG **GPGPG**GIHDNCAVLPTHAEC**GPGPG** LCLLAWHNGKQQYED**KK**KEPAALH DKD**KK**YPYVTL**KK**EPAALHDKDPRL DLNT**KK**QDLNTSAGYPYVTLGI**KK**K SLCLLAWHNGKQQYE**GGGS**	Non allergen	0.7140	0.626

### 3.8 Evaluation of the constructed vaccine

**Figure 4:**
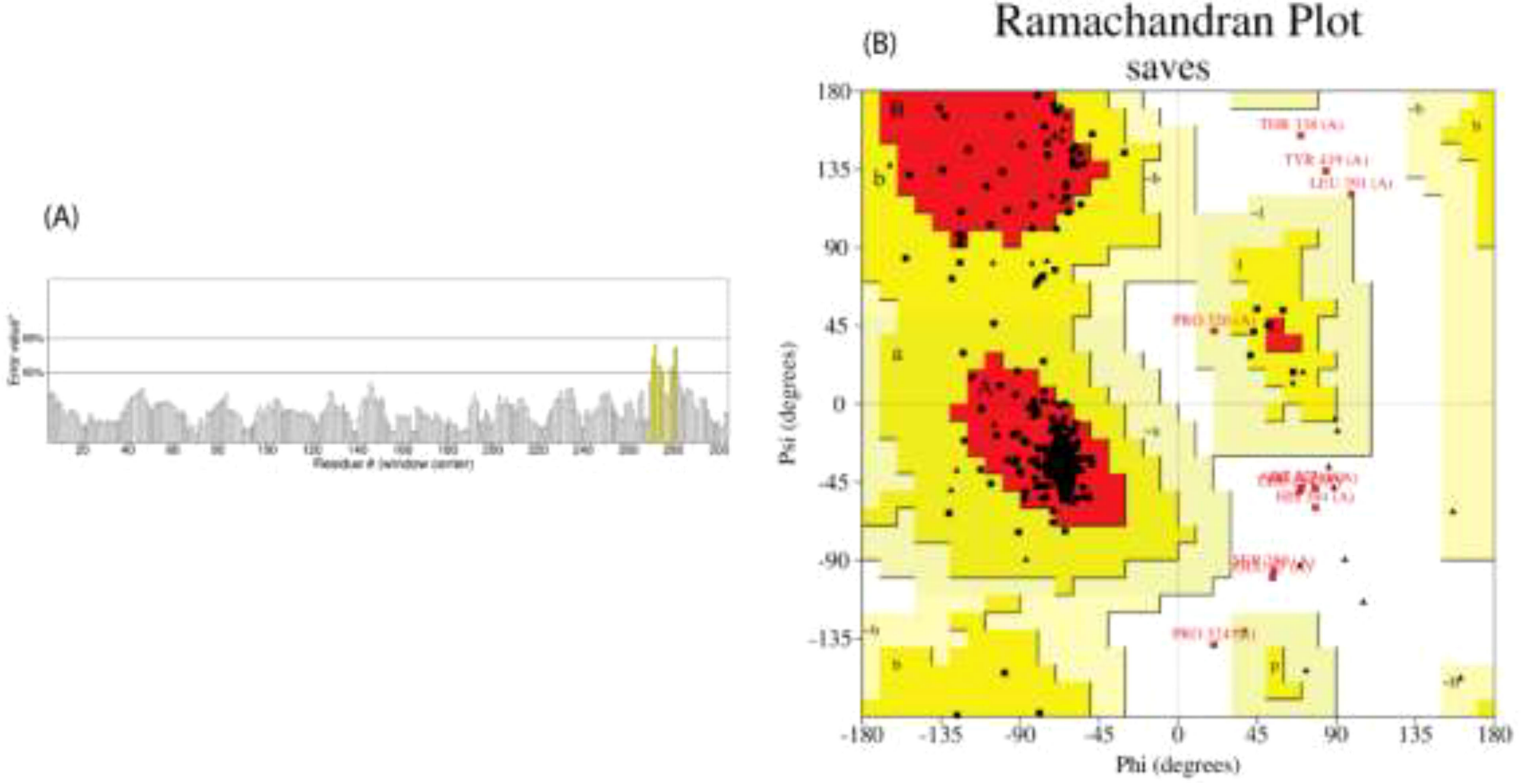
We assessed the predicted model’s quality using multiple validation techniques for the V2 vaccine. **(A)** Error value plot indicating residue-specific quality, with an overall ERRAT quality factor of 91.921, highlighting high model quality; **(B)** Ramachandran plot, generated using the PROCHECK server, shows the distribution of phi (ϕ) and psi (ψ) angles for residues to evaluate stereochemical quality. Favored regions are in dark red, allowed regions in yellow, and outliers.

As per the server’s findings, the data supported the robust antigenicity and non-allergenicity of the vaccine candidates (**Table 7**). All three vaccines exhibited a solubility value exceeding 0.550, surpassing the standard threshold of 0.45, indicating promising solubility potential. All proposed vaccines were determined to be non-toxic. To ensure a strong immune response post-vaccination, it is crucial for the vaccine design to be highly antigenic and immunogenic while remaining safe and non-allergenic (65). We assessed the quality of the predicted model using various validation methods for the V2 vaccine (**Figure 4**). The ERRAT analysis yielded a high-quality factor of 91.921, indicating robust model accuracy with minimal deviations. The Ramachandran plot, generated using the PROCHECK server, further assessed the stereochemical quality of the model. Most residues were positioned within favored and allowed regions, confirming proper folding and stability. Some residues, such as THR 338, TYR 419, and LEU 391, were located in outlier regions, suggesting potential areas for further refinement. These results collectively validate the structural quality and reliability of the modeled vaccine candidate. The physicochemical characteristics of the three vaccine constructs, V1, V2, and V3, highlight their structural and functional properties crucial for immunogenicity, no toxic and stability. V1 consists of 405 amino acids with a molecular weight of 41292.42 Daltons and a theoretical isoelectric point (pI) of 5.59. It exhibits an instability index of 25.53, indicating moderate stability, and an aliphatic index of 76.77, suggesting a high proportion of aliphatic side chains. Evaluation of physio-chemical properties predicted the vaccines to be stable, hydrophilic, and highly soluble upon over-expression in *E. coli* (**Table 8**).

**Table 8:** Physicochemical properties of vaccine construct.

Vaccine construct	Number of amino acids	Molecular Weight (Dalton)	Theoretical pI	Half-life (In *Escherichia coli*, *in vivo*)	Instability index	Aliphatic index	Grand average of hydropathicity
V1	405	41292.42	5.59	>10 hours	25.53	76.77	-0.332
V2	480	50028.13	8.32	>10 hours	33.53	71.79	-0.582
V3	320	33013.13	8.86	>10 hours	30.41	64.44	-0.592

### 3.9 Vaccine structure prediction and 3D structure validation

**Figure 5:**
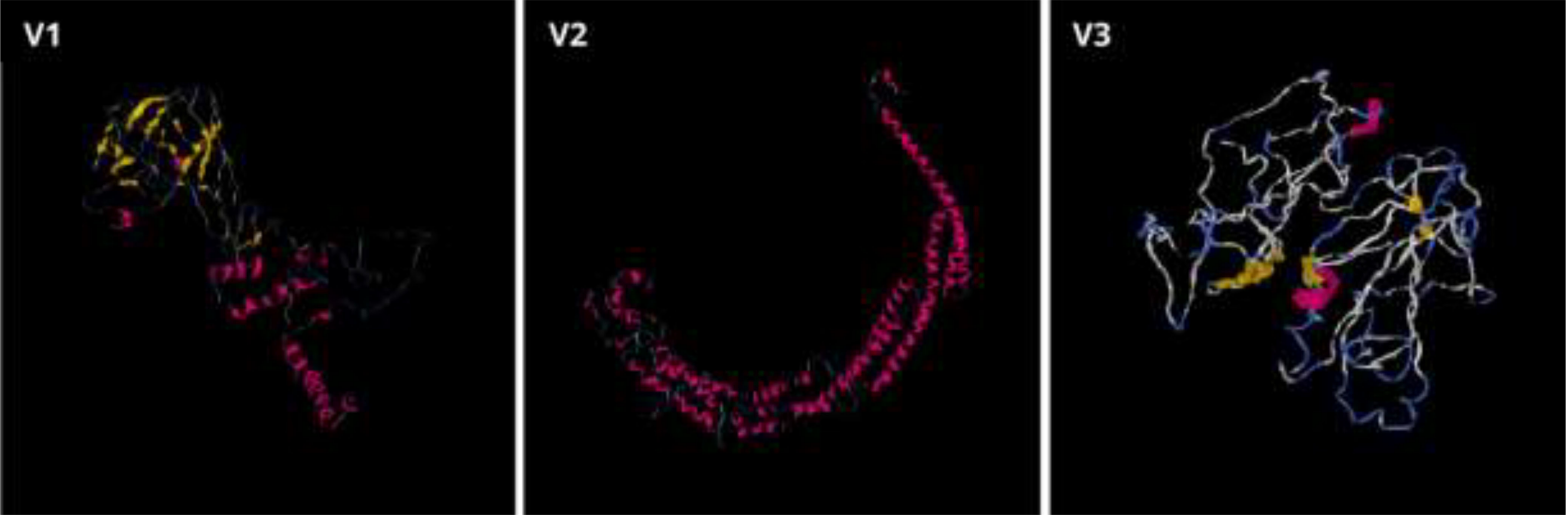
Three-dimensional structures for the vaccine proteins V1, V2, and V3 were produced through the I-TASSER server.

The secondary structures indicated that 46.67% were random coils, 27.5% were helices, 16.3% were extended strands, and 9.38% were turns for V1 (**Table 9**). In V2, the alpha-helix region comprised the highest percentage of amino acids (39.79%), while the beta turn structure had the lowest percentage (5.83%). Furthermore, the I-TASSER tool was utilized to generate five distinct models for each vaccine protein, thoroughly exploring various structural configurations (**Figure 5**). Afterward, the GalaxyRefine server was employed for refinement, producing a series of five altered 3D models for each vaccine. The selection of the best models involved assessing them through the Ramachandran plots on the PROCHECK website. As per the Ramachandran Plot results, V2 exhibited 91.9% of its residues in the most favorable locations, with 7.3% falling within permitted regions (**Table 10**). A widely accepted standard dictates that a 3D model is considered reliable when over 90% of its residues are in the most favorable locations (66). As per the ERRAT and Verify 3D findings, the improved structure of V2 demonstrated an overall quality factor of 90.96% and 80.42%, respectively. In the end, V2 was chosen for additional analysis because it had the highest quality score.

**Table 9:** Secondary structural analysis of vaccine constructions.

Vaccine construct	Beta Sheet/Extended strand	Alpha Helix	Random coil	Beta turn
V1	16.30%	27.65%	46.67%	9.38%
V2	12.29%	39.79%	42.08%	5.83%
V3	20.00%	10.94%	60.31%	8.75%

**Table 10:** Assessment of the structural integrity took place both before and after utilizing the GalaxyRefine server for refinement.

Vaccine	Ramachandra Plot (Residues in the most favorable region)		Verify 3D (Compatibility of an atomic mode)		ERRAT (Overall quality factor)	
	Before (%)	After (%)	Before (%)	After (%)	Before (%)	After (%)
V1	58.0	79.0	80.49	81.48	75.79	75.731
V2	71.1	91.9	72.29	80.42	85.85	90.96
V3	40.4	68.1	92.81	96.88	66.66	35.10

### 3.10 Estimation of B-cell epitopes and disulfide-based vaccine prediction

**Figure 6:**
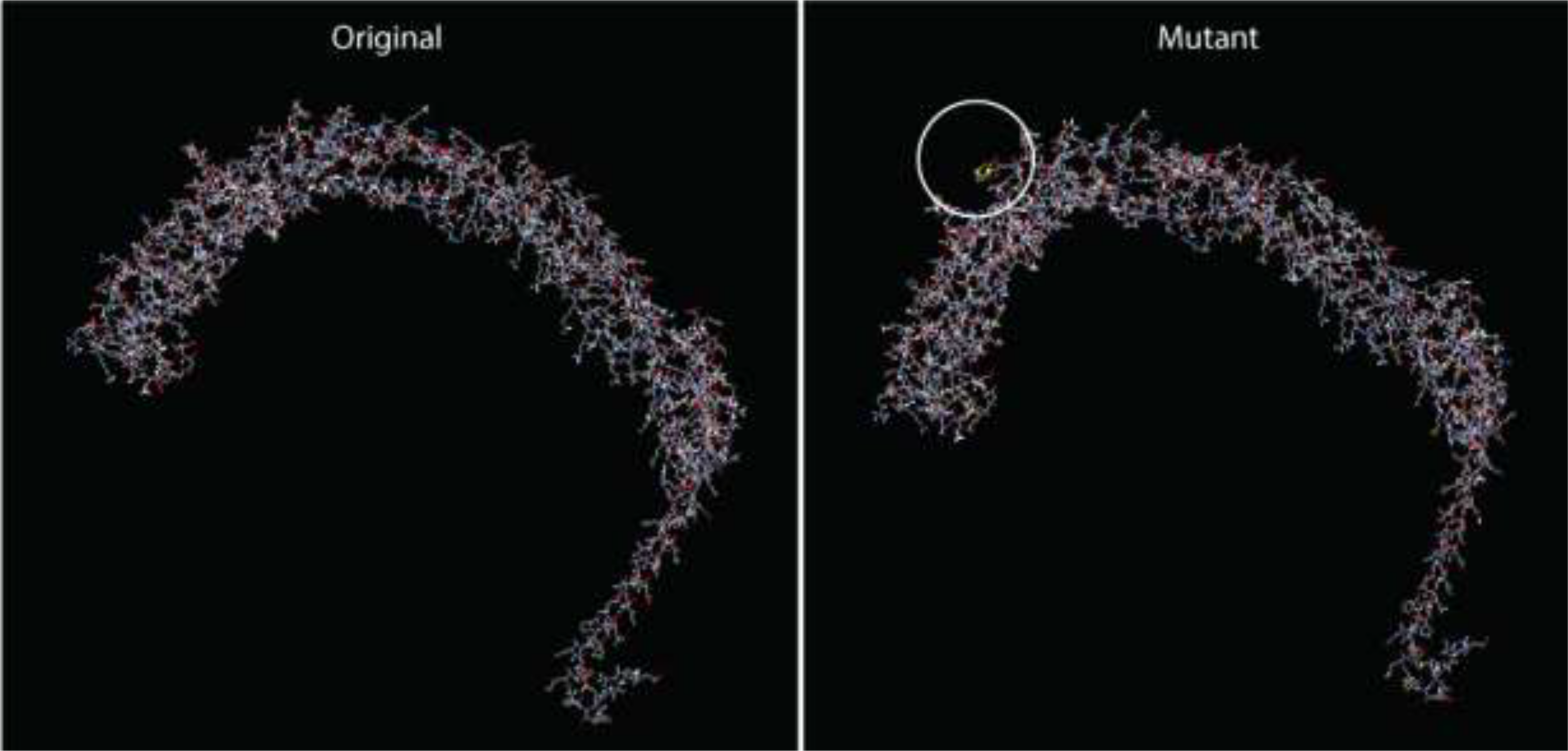
Disulfide engineering in the final vaccine construct (V2), depicting the modified residue pair.

Twenty-three potential disulfide bonding sites in the V2 vaccine candidate were identified using the DbD2 server. Only one residue pair satisfied the predetermined criteria based on bond energy and χ3 features. The bond energy and the χ3 angle needed to fall below 2.2 kcal/mol, and the χ3 angle needed to fall between -87 and +97 degrees. According to these criteria, no sequence was deemed acceptable for disulfide production (**Supplementary Table 8**). However, a pair of mutations were produced on the residue pair CYS361-ASP371, responsible for the production of disulfide bonds (**Figure 6**). The protein domain has an adequate quantity of B-cell epitopes crucial for humoral immunity since B-lymphocytes secrete cytokines and antibodies to neutralize foreign antigens. Utilizing the updated 3D structure V2, the Ellipro server forecasted the conformational B-cell epitopes. The scores for these linear epitopes ranged from 0.514 to 0.804, and their residue numbers ranged from 4 to 126 (**Supplementary Tables 9**, **10**).

### 3.11 Molecular docking and simulation

**Figure 7:**
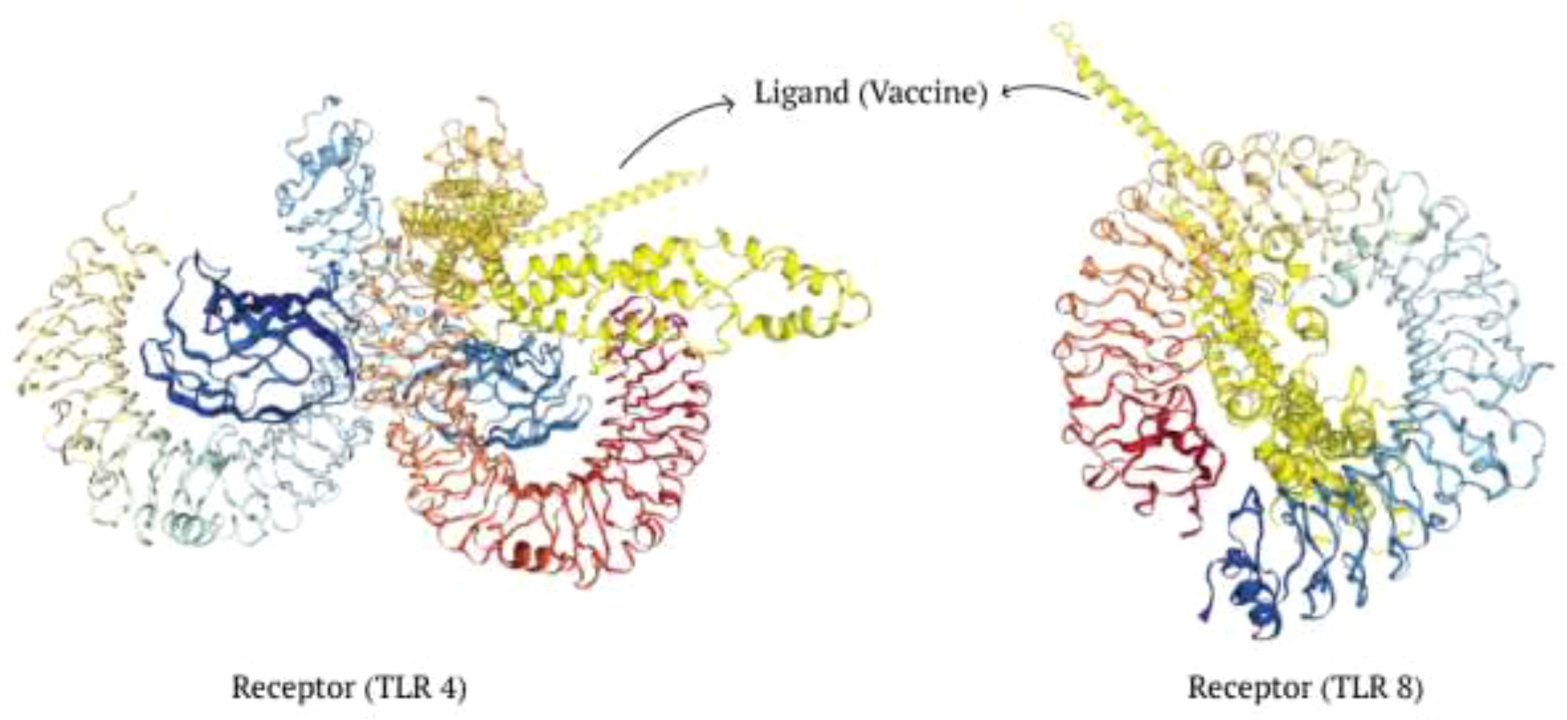
Investigation of interaction between the ligand and receptors.

The antigenic molecule must recognize and bind with the appropriate immune receptor molecule to initiate the host immune response. A thorough analysis was conducted to investigate the binding affinity of the vaccine to antigenic receptors such as TLR4 and TLR8. The effectiveness of peptide-based vaccines may be enhanced by using TLR8 agonists, which activate dendritic cells and trigger Th1 and CD8^+^ T cell responses (67). Protein-protein docking was performed using the Hdock server, selecting only the best structure with the lowest binding energy for further study (**Table 11**). The docking score for TLR4 docking was -257.32, whereas the docking score for TLR8 docking was -296.15. Consequently, the most effective docking complex was visualized with the Pymol tool (**Figure 7**).

**Table 11:** Protein-protein docking score of V2 by Hdock server.

Ligand	Receptor	Docking Score	Confidence Score	Ligand rmsd (Å)
V2	TLR4 (PDB ID: 4G8A)	-257.32	0.8953	243.73
	TLR8 (PDB ID: 3W3M)	-296.15	0.9490	282.32

**Figure 8:**
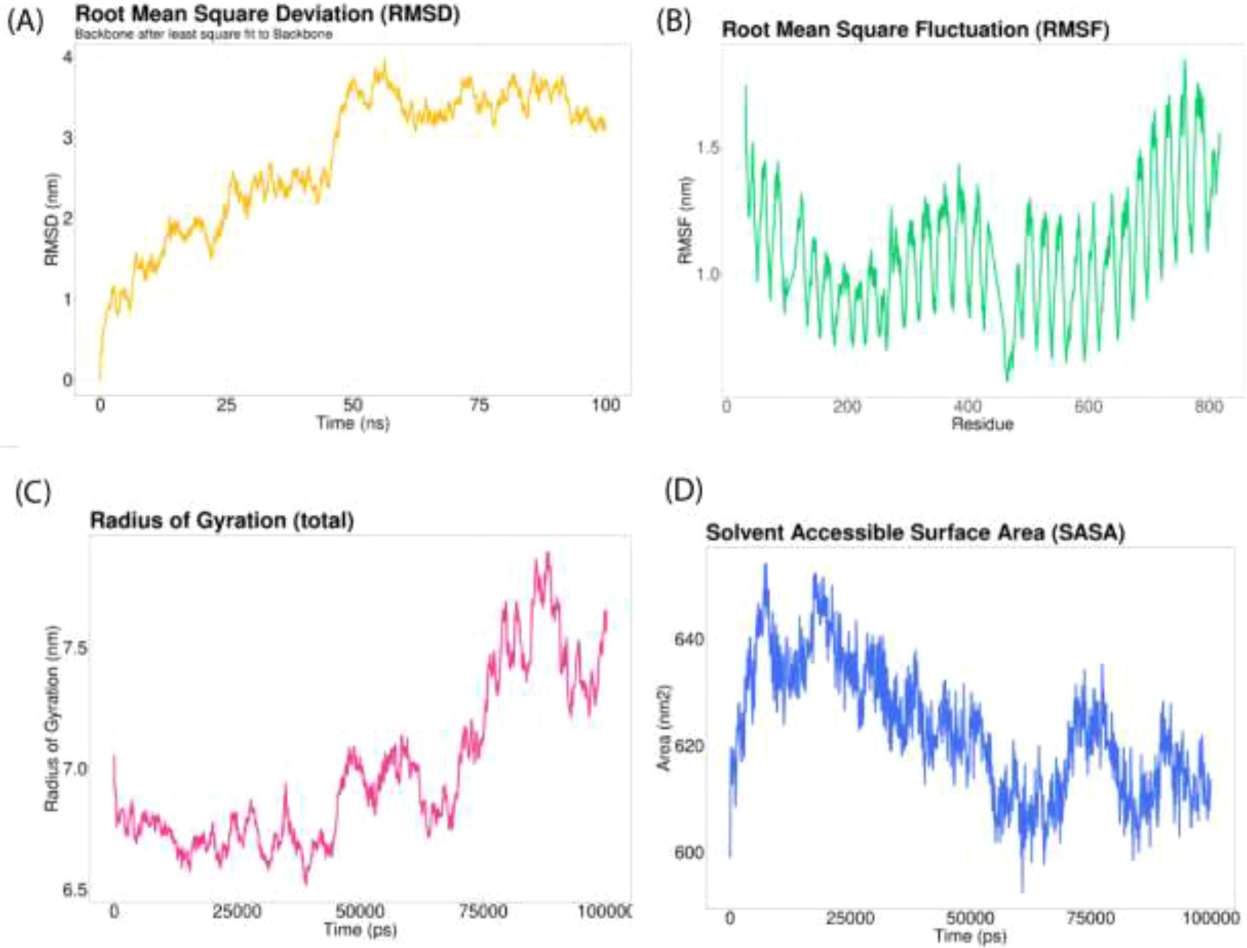
Molecular dynamics simulation of 100 ns. **(A)** The RMSD of the docked complex serves as an indicator of the stability of the vaccine interaction; **(B)** The RMSF provides insight into the flexibility and fluctuation of amino-acid residues; **(C)** Monitoring the time-dependent changes in the radius of gyration; **(D)** Average value of the SASA during a molecular simulation.

The GROMACS tool was employed to simulate the Vaccine-TLR8 complex for 100ns, studying the dynamic behavior and interactions between the components. The computation of root mean square deviation (RMSD) was carried out to assess the system’s stability, with an increased RMSD value indicating a shift in the protein conformation (**Figure 8A**). Between approximately 50 ns and 60 ns, a notable alteration in the vaccine-receptor complex was observed. Subsequently, the overall RMSD value remained consistently elevated throughout the simulation without significant fluctuations. Root mean square fluctuation (RMSF) was employed to assess the flexibility of different protein regions, with increased RMSF indicating greater flexibility of amino acids (**Figure 8B**). Throughout the simulation, the mobility of the receptor exhibited frequent changes. The radius of gyration (Rg) served as a metric for assessing the compactness of a protein, with a consistently stable Rg value indicating a well-folded and stable protein structure (**Figure 8C**). The Rg value exhibited an increase throughout the simulation. Solvent-accessible surface area (SASA) was employed in MD simulations to forecast the stability of the hydrophobic core in proteins, with a higher SASA value suggesting an increased likelihood of protein destabilization (**Figure 8D**). The SASA value for the vaccine-receptor complex gradually decreased over time in the simulation.

### 3.12 Adaptation of codon, in silico cloning and immunological response triggered by vaccine candidates

**Figure 9:**
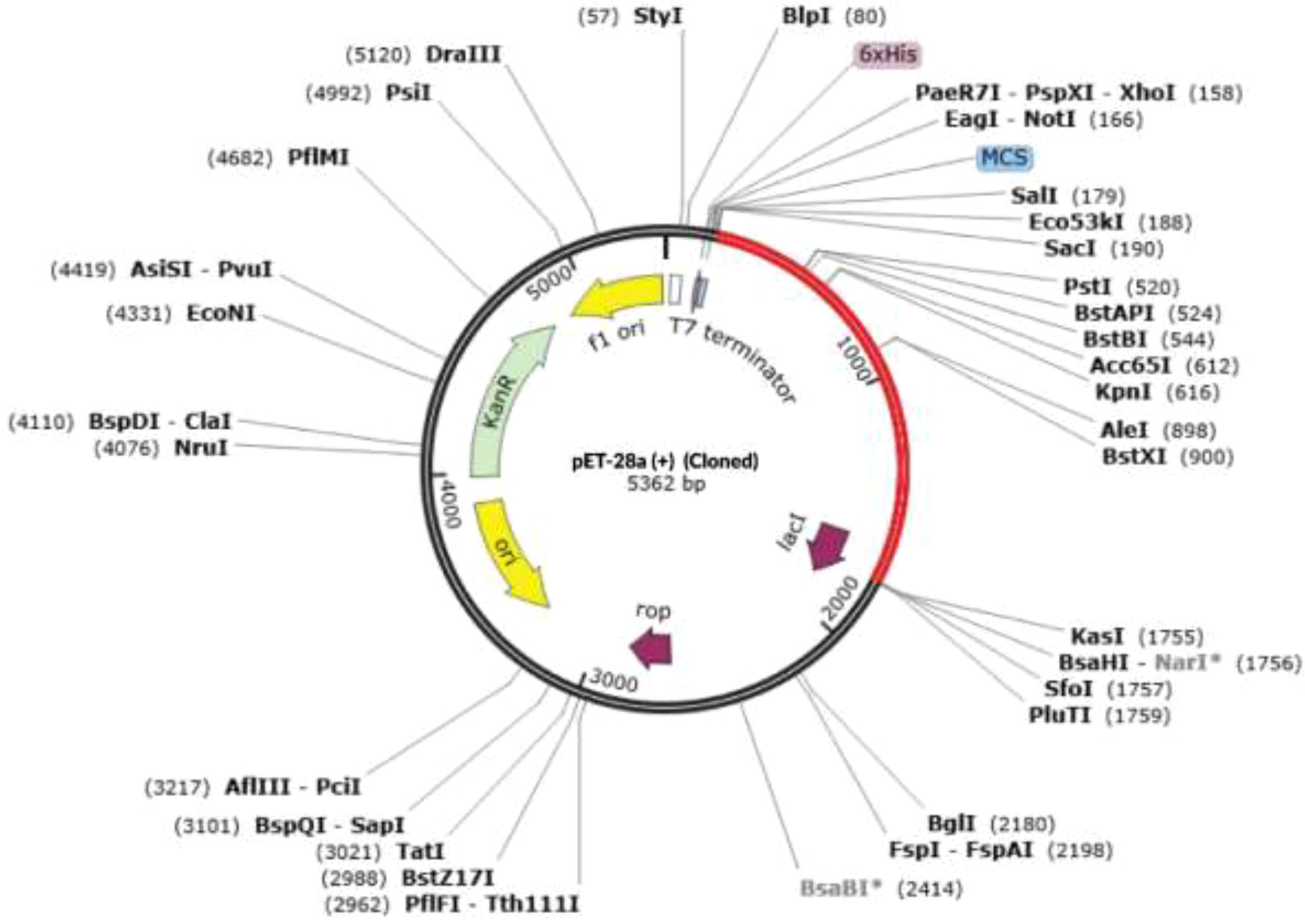
The virtual cloning of the optimized final vaccine construct (V2) into the pET-28a (+) expression vector. Restriction digestion of the vector pET28a(+) and construct V2 was performed using Eco53KI (188) and SfoI (1757), with the sites highlighted in red. The inserted fragment is also marked in red.

**Figure 10:**
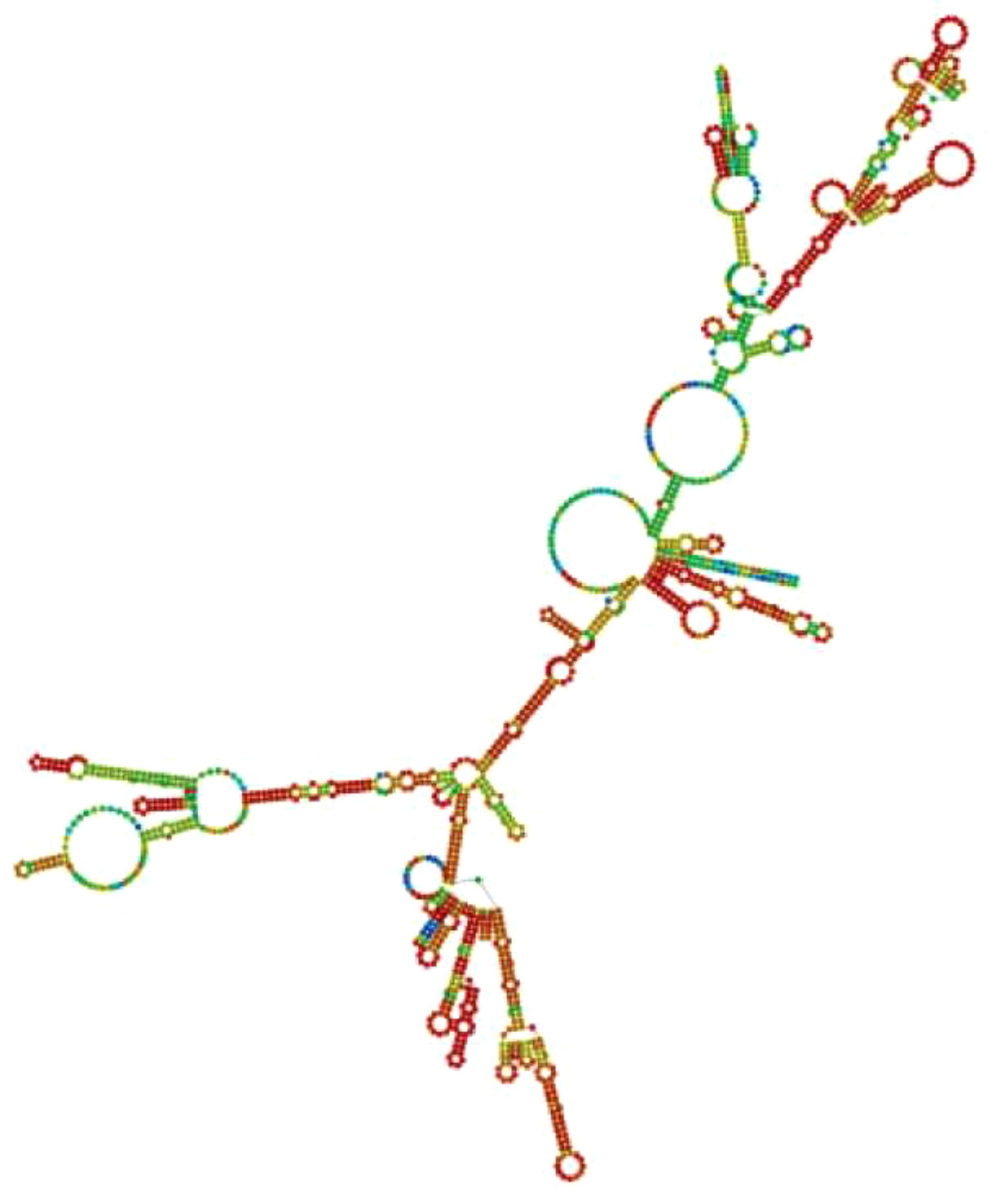
Anticipated secondary structure of the mRNA for the developed vaccine construct.

The JCatsite tool was employed to reverse-translated and codon-optimize the final vaccine protein sequence (V2). The optimized codon sequence for enhanced output from *E. coli* (strain K12) had a length of 1440 nucleotides. The sequence was deemed suitable with a Codon Adaptation Index (CAI) of 0.99, indicating the frequent use of codons, and an adjusted GC content of 54.58. The restriction sites were selected based on the common restriction endonucleases present in both the vector and the vaccine candidate. The optimized codon sequence for enhanced expression in *E. coli* (strain K12) was synthesized to include these specific sites, ensuring precise and efficient insertion into the expression vector pET28a(+). To ensure successful cloning, the anticipated V2 DNA sequence was inserted into pET-28a(+) carrier plasmids between the Eco53KI (188) and SfoI(1757), restriction sites (**Figure 9**). The overall goal of this optimization was to achieve robust and reliable production of the target protein in the *E. coli* expression system, facilitating subsequent purification and application in vaccine development. The resulting vaccine sequence underwent RNA folding, forming a secondary structure with a minimum free energy of -494.90 kcal/mol (**Figure 10**).

**Figure 11:**
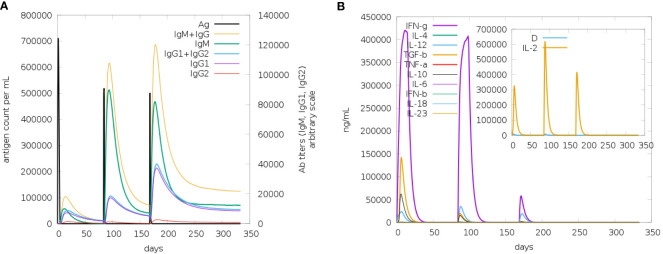
The antigen counts per cell **(A)** showed a significant stimulation of antibody synthesis following the administration of antigens. This indicates that the vaccine effectively prompted the immune system to produce antibodies, a crucial aspect of developing immunity. In panel **(B)**, the degree of cytokine expression was analyzed. Cytokines are essential for coordinating the immune response, and their elevated levels post-vaccination suggest that the vaccine successfully triggered the necessary signaling pathways to enhance the immune defense. The increase in cytokine levels is indicative of an active and responsive immune system, further supporting the vaccine’s potential efficacy.

The immune simulation study for the designed vaccine V2 was conducted using the C-ImmSim program. Three injections were administered at time steps of 1, 252, and 504, respectively, each representing an 8-hour real-life interval. This simulated the typical vaccination schedule, with doses spaced to allow for optimal immune response development. The results from **Figure 11** demonstrated a robust immune response to the vaccine. The antigen counts per cell (**Figure 11A**) showed a significant stimulation of antibody synthesis following the administration of antigens. This indicates that the vaccine effectively prompted the immune system to produce antibodies, a crucial aspect of developing immunity. In **Figure 11B**, the degree of cytokine expression was analyzed. Cytokines are essential for coordinating the immune response, and their elevated levels in post-vaccination suggest that the vaccine successfully triggered the necessary signaling pathways to enhance the immune defense. The increase in cytokine levels is indicative of an active and responsive immune system, further supporting the vaccine’s potential efficacy.

**Figure 12:**
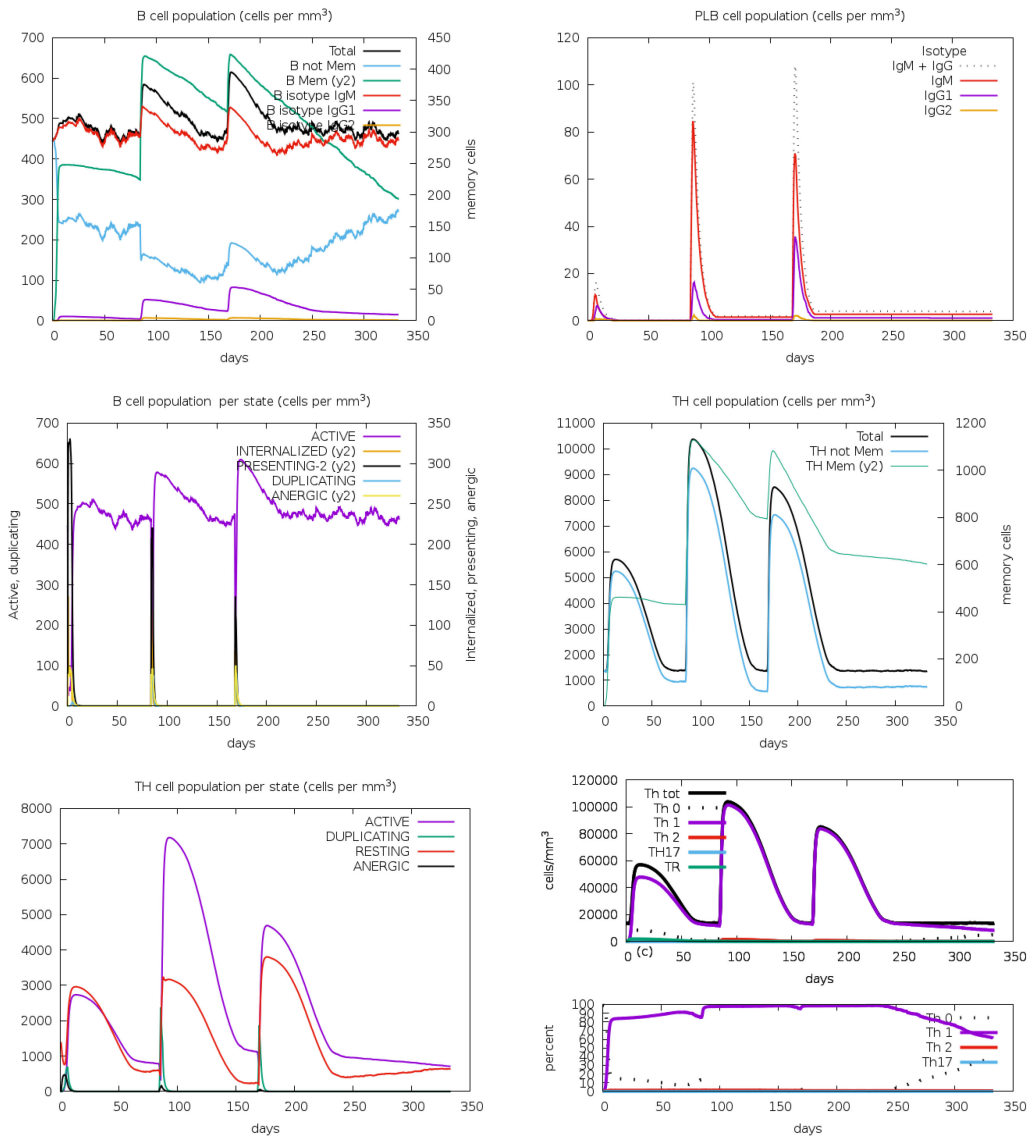
The immune simulation study for the designed vaccine V2 provided insightful results regarding the B-cell, plasma B (PLB) cell, and T-helper (TH) cell population concentrations. These results were analyzed through the C-ImmSim program, which simulated the immune response to the vaccine following three injections.

The B-cell population showed a substantial increase during post vaccination, indicating effective stimulation of B-cell differentiation and proliferation. This rise suggests a robust humoral immune response, essential for pathogen recognition and neutralization. Plasma B cells, responsible for antibody secretion, also increased markedly, signifying that the vaccine promotes B-cell maturation into antibody-producing cells (**Figure 12**). This increase corresponds with elevated antibody levels, reinforcing the vaccine’s effectiveness. Additionally, the T-helper (TH) cell population displayed a positive trend, indicating successful activation. TH cells are crucial for activating B-cells, cytotoxic T-cells, and macrophages, ensuring a coordinated and comprehensive immune response.

**Figure 13:**
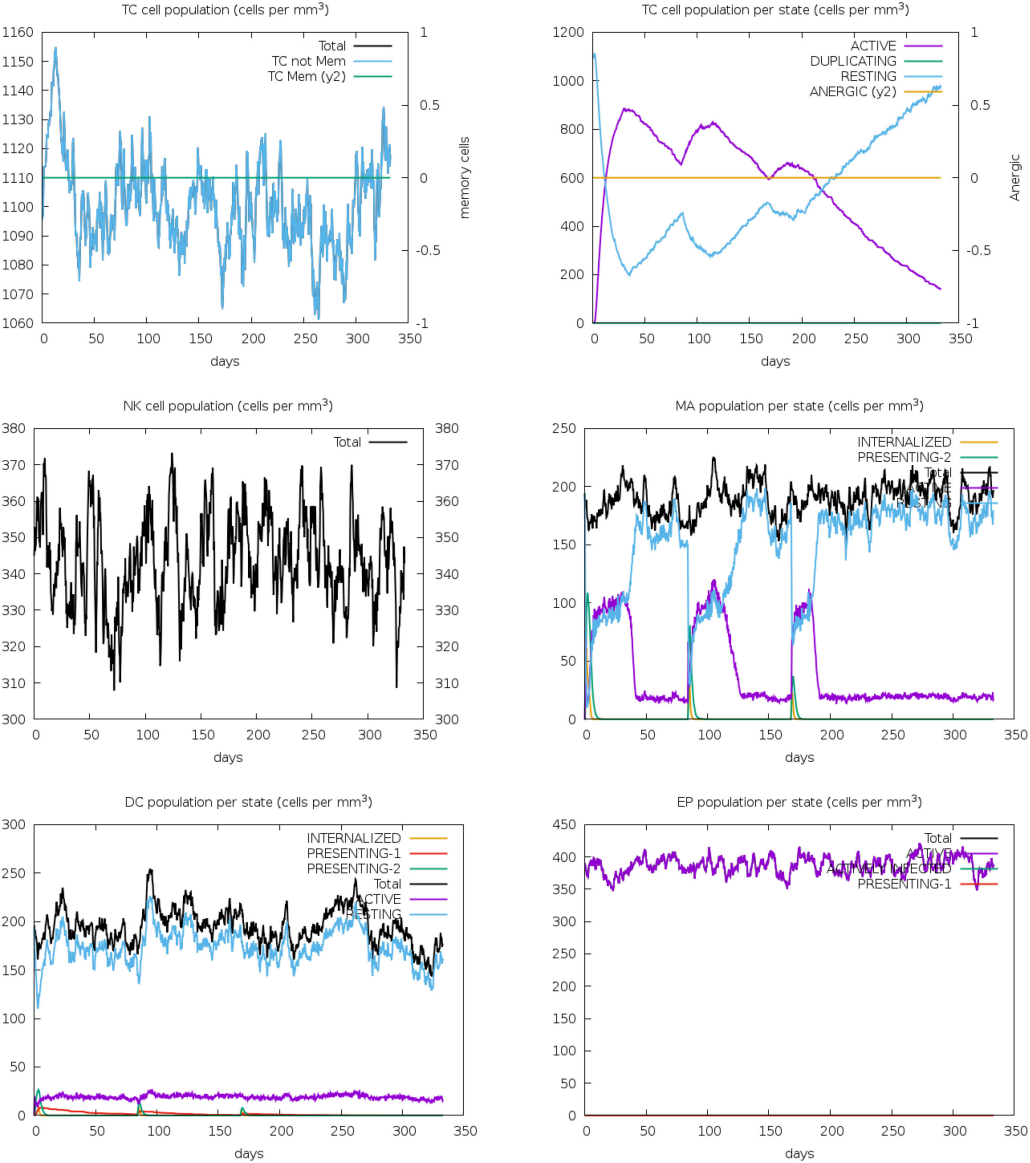
The immune simulation study for vaccine V2 demonstrated substantial increases in the population densities of T-cells, NK cells, macrophages, dendritic cells, and effector cells. These findings suggest that the vaccine effectively stimulates a comprehensive immune response, engaging both the innate and adaptive arms of the immune system.

Following vaccination, there was a significant increase in the population densities of various immune cells. T-cells, both cytotoxic (CD8^+^) and helper (CD4^+^), showed notable growth, indicating effective activation and a robust cell-mediated immune response. NK cells, crucial for early viral defense, also increased, suggesting enhanced innate immunity (**Figure 13**). Macrophages, responsible for phagocytosis and cytokine production, saw a rise in numbers, highlighting the vaccine’s ability to engage these cells for pathogen clearance. Dendritic cells, essential for antigen presentation and T-cell priming, also increased, ensuring a targeted immune response. The overall increase in effector cells, including cytotoxic T-cells and activated macrophages, signifies strong immune activation, equipping the immune system to neutralize and eradicate the pathogen effectively.

## 4 Discussion

Vaccination is the most effective method now available for preventing against infectious diseases, particularly viral infections. Researchers have been diligently pursuing the development of more affordable, time-efficient, and secure vaccines to combat infectious diseases, especially viral infections. However, addressing specific challenges remains crucial, particularly in the case of human rhinoviruses, which are responsible for the common cold and upper respiratory tract infections, particularly in young children and newborns (68). The prevalence of HRV infections is compounded by the extensive genetic variability and a broad spectrum of serotypes, posing formidable obstacles to vaccine development and efficacy. Our study adopted a reverse vaccinology approach, focusing on multi-epitope vaccines in response to this exigency. These vaccines incorporate immunogenic components of pathogens to target specific antigenic epitopes, resulting in higher efficiency with minimal adverse effects. This strategic approach aims to overcome the challenges posed by HRV’s genetic diversity, offering a promising avenue for developing a more effective and tailored vaccine against these common respiratory infections.

In this investigation, we retrieved three viral proteins (VP1, VP2, and 2C) of HRV-C from the NCBI database using a literature search to ensure the selection of the most relevant proteins as antigens. The abundance of T-cells and neutralizing epitopes in structural proteins can provoke substantial cellular and humoral immune responses, rendering them optimal targets for peptide vaccines (69, 70). Additionally, non-structural proteins perform critical functions in viral transmission and remain highly durable (71). The rhinovirus 2C protein exhibits RNA helicase activity, a crucial function for viral replication. It helps unwind the viral RNA during replication and transcription processes, facilitating the synthesis of viral RNA. Proteins were chosen based on allergic reaction, topology, and antigenicity score since these variables correlate with immune response. Different databases were employed to predict T and B cell epitopes, and their efficacy as a vaccine candidate was assessed. T-cell epitope-based vaccines elicit robust immune responses against viruses, with B-cells playing a pivotal role through cytokine release to prevent disease occurrence and spread (72, 73). The release of IFN-γ by helper T lymphocytes (HTLs) is essential for the body’s antiviral response. Five HTL epitopes were selected based on high antigenicity, ability to stimulate IFN-γ, and potential to induce IL-4.

Our analysis found 68% global coverage for the selected T-cell epitopes and HLA alleles. A previous study reported that 60-80% of the population possesses immunity to the Ebola virus (74). We performed molecular docking to assess the binding interactions between HLA molecules and the selected T-cell epitopes. All the epitopes demonstrated a notable level of binding affinity with their corresponding alleles.

After a meticulous assessment of conservancy, antigenicity, allergenicity, topology, toxicity, and cytokine-inducing potential, epitopes were chosen for the vaccine. Additionally, appropriate linkers were selected to enhance the cohesiveness and stability of the vaccine construct. Prior studies suggest that augmenting the N-terminal of the vaccine fusion protein with EAAAK enhances its bioactivity (75).

Adjuvants in vaccine design could influence immunity, antigen fabricating, stability, and durability to better protection against infection (76). In order to determine the suitable adjuvant, an evaluation was conducted on three peptides: a 50 S ribosomal protein L7/L12, heparin-binding hemagglutinin, and beta-defensin-3. Numerous studies have shown the usefulness of beta-defensin adjuvant as an immune stimulator against various pathogens (77). During this investigation, three distinct vaccines were formulated, employing an approach where each epitope was individually linked with appropriate linkers and adjuvants. The vaccines exhibited non-allergic, antigenic properties, and water solubility, according to the results of the vaccination evaluations. Based on an assessment of antigenicity and solubility, it was determined that construct V3 exhibited a higher level of excellence compared to constructs V1 and V2. Furthermore, the three construct vaccine proteins showed stability, thermostability, and hydrophilicity. Proteins with a molecular weight of less than 110 kDa, allowing for rapid purification, are favored as prime candidates for vaccine development (78). The vaccine’s 3D structure was crafted with the I-TASSER server, emphasizing V2 due to its outstanding performance through various servers.

In a wide variety of biological and therapeutic applications, increasing protein stability is of the most significant relevance and conveys the highest value (79). In order to improve the thermal stability and characteristics of the protein, disulfide engineering, introducing mutations on CYS361-ASP371, improved thermal stability. B-lymphocytes play a pivotal role in the production of antibodies and cytokines, which are essential for the neutralization of foreign antigens. In addition to helping with more accurate prediction, linear B-cell epitope prediction may facilitate a quicker and less costly vaccine design process (80).

Docking evaluated the binding affinity of produced V2 and cattle immunoreceptors. Prioritizing the lowest global energy resulted in -257.32 and -296.15 for TLR4 and TLR8, respectively. The GROMACS tool simulated the docked V2-TLR8 complexes over 100 nanoseconds. MD simulations were used to study the stability and dynamics of the vaccine-receptor complex. The RMSD analysis indicated a notable conformational change in the complex between 50 and 60 nanoseconds, suggesting a dynamic structural adjustment. Subsequently, RMSD values stabilized at an elevated level, suggesting overall stability without notable fluctuations. RMSF analysis highlighted varying degrees of mobility within receptor regions throughout the simulation, underscoring the dynamic nature of the receptor. Radius of gyration results indicated a progressive increase in protein compactness over the simulation period, suggesting a tendency towards a more folded conformation. In contrast, SASA analysis revealed a gradual decline in hydrophobic core stability, potentially implying alterations in the protein’s interactions with its environment. These results imply a nuanced interplay among stability, flexibility, and structural changes within the vaccine-receptor complex throughout the MD simulation. This provides valuable insights into its dynamic behavior and potential implications for vaccine design and optimization strategies.

The solubility of recombinant proteins over expressed in *E. coli* is critical for conducting biochemical and functional experiments. Codon adapting is a crucial step in vaccine design, expressing foreign genes efficiently. Following *E. coli* strain K12-specific adaptation of the suggested vaccine design V2 via reverse transcription, cloning, and heterologous expression using the pET28a(+) vector, the process concludes with the JCat server. The practical implementation of in silico cloning suggests the potential for large-scale vaccine production in *E. coli* (81).

The vaccine candidate imitated the typical immunological response to viral infections by generating an immune reaction against the Rhinovirus antigen, as immunological simulation data shows (**Figures 9**–**11**). The results demonstrated significant antibody synthesis and elevated cytokine levels, indicating a robust immune response. Post-vaccination, the B-cell population increased substantially, showing effective B-cell differentiation and proliferation, essential for humoral immunity. Plasma B (PLB) cells and T-helper (TH) cells also increased, ensuring a coordinated immune response. The population densities of various immune cells, including cytotoxic (CD8^+^) and helper (CD4^+^) T-cells, NK cells, macrophages, and dendritic cells, showed notable growth. This indicates effective activation of both innate and adaptive immunity, ensuring the immune system is well-equipped to neutralize and eradicate the pathogen. However, rigorous experimental validations are necessary to transition from in-silico design to a clinically viable and effective vaccine.

## 5 Conclusion

The design of a multi-epitope vaccine targeting human rhinovirus C (HRV-C) showcases a promising strategy for addressing this significant respiratory pathogen. By incorporating adjuvants and suitable linkers alongside predicted T-cell and B-cell epitopes, we enhanced the vaccine’s immunogenicity. Our findings demonstrate that the developed vaccine candidates fulfill all essential criteria for antigenicity, solubility, allergenicity, and favorable physicochemical properties. Molecular docking and molecular dynamics simulations conducted on TLR2 and the leading vaccine candidate (V2) further confirmed the stability of the vaccine complex and its binding affinity. Additionally, the codon optimization yielded a highly favorable Codon Adaptation Index (CAI) value, indicating strong potential for successful *in vivo* expression. Thus, it can be concluded that the developed multi-epitope vaccine candidate can effectively elicit both innate and adaptive immunity. However, to transition from theoretical efficacy to practical application, further *in vivo* tests are necessary to evaluate the vaccine’s effectiveness and safety in a vaccination model. This research lays a crucial foundation for future HRV-C vaccine development and emphasizes the importance of rigorous testing to validate our findings.

### 5.1 Limitations and recommendation

This study, while promising, has several limitations. The epitope predictions and vaccine design are based on computational tools, which may not always be accurate. HRV-C’s genetic variability could impact the vaccine’s effectiveness across different serotypes. The study lacks experimental validation, relying solely on in silico methods. The actual *in vivo* effects of adjuvants and linkers could differ from predictions. Challenges in vaccine expression and purification need experimental assessment. The long-term immunogenicity and protective efficacy are not addressed, necessitating longitudinal studies. Finally, transitioning from design to clinical application involves rigorous regulatory and ethical considerations. Comprehensive experimental studies and clinical trials are essential to validate the vaccine’s safety and efficacy.

## Data Availability

The original contributions presented in the study are included in the article/**Supplementary Material**. Further inquiries can be directed to the corresponding authors.
