# Supplementary material for: Designing a multi-epitope vaccine candidate against human rhinovirus C utilizing immunoinformatics approach
Source: Front Immunol. 2025 Jan 7;15:1364129. doi: 10.3389/fimmu.2024.1364129 (PMC11747413; doi:10.3389/fimmu.2024.1364129)
Supplement: Supplementary file 1 [file Table1.doc]

Program		Purpose	
NCBI	https://www.ncbi.nlm.nih.gov/	Proteins sequences of HRV-C retrieved  	
TMHMM 	https://services.healthtech.dtu.dk/services
/TMHMM-2.0/	Topology analysis of the protein sequences	
VaxiJen v2.0	https://www.ddg-pharmfac.net/vaxijen/
VaxiJen/VaxiJen.html	Antigenicity analysis of protein 	
ExPASy	http://web.expasy.org/protparam	Physicochemical properties of analysis	
BLASTp	https://blast.ncbi.nlm.nih.gov/Blast.cgi?
PAGE=Proteins	Conserved area of proteins to the following viruses	
Clustal Omega server	https://www.ebi.ac.uk/Tools/msa/clustalo/	Multiple Sequence Alignment of protein	
IEDB 	https://www.iedb.org/	T-cell and B-cell epitopes identification, population coverage,   epitope conservancy 	
ABCpred	http://crdd.osdd.net/raghava/abcpred/	B-cell epitopes identification	
IL4pred	http://crdd.osdd.net/raghava/il4pred/	Ipitopes capable of inducing IFN-ã	
PEP-FOLD	https://mobyle.rpbs.univ-paris-diderot.fr/
cgi-bin/portal.py#forms::PEP-FOLD3	Peptide tertiary structure prediction	
AllerTOP 	https://www.ddg-pharmfac.net/AllerTOP/	Allergenicity analysis	
ToxinPred	https://webs.iiitd.edu.in/raghava/toxinpred/
multi_submit.php	Toxicity anticipation	
Protein-Sol	https://protein-sol.manchester.ac.uk/	solubility of the vaccine constructs	
PRISPRED	http://bioinf.cs.ucl.ac.uk/psipred	assess the secondary structures of vaccine constructs	
I-TASSER	https://zhanggroup.org/I-TASSER/	tertiary structures of the vaccines	
GalaxyRefine	https://galaxy.seoklab.org/cgi-bin/submit.cgi?
type=REFINE	Refining of the three-dimensional structures	
PROCHECK	https://saves.mbi.ucla.edu/	Validation of the three-dimensional structures	
DbD2	http://cptweb.cpt.wayne.edu/DbD2/	create new disulfide links in proteins	
Ellipro server	http://tools.iedb.org/ellipro/	predict the vaccination's discontinuous epitopes	
Hdock	https://hdock.phys.hust.edu.cn/	Protein-protein docking 	
C-ImmSim immune server	https://kraken.iac.rm.cnr.it/C-IMMSIM/	determining the immunogenic profile of the Vaccine	
JCat	http://www.jcat.de/	Codon adaptation	
RNAfold	http://rna.tbi.univie.ac.at/cgibi/RNAWebSuite/
RNAfold.cgi	RNA secondary structure	
Table 1. Servers were used in this study


Table 2. Antigenicity score and topology of identified conserved regions
Protein	Conserved regions	Antigenicity score	Allergenicity	Topology	Remarks	
VP1	MTNPALMSPSIEACGFSDRLKQITIGSSTITTQ
DTLNT	0.5589	Non-allergen	Outside	Selected	
	MGAQVSKQNVGSHESGISASSGSVIKYFNINY
YKDS
ASSGLSKQDFSMDPEKFTKP	0.4385	Allergen	Inside	Not-
Selected	
	PYINCVPMDSMLKHNN	0.6791	Non-allergen	Inside	Not-
Selected	
	TAGGENHVAFTDLRPY	1.0942	Non-allergen	Outside	Selected	
	NAQIRKKLELFTYVRFDLE	 0.7074	Non-allergen	Inside	Not-
Selected	
	VPMAALEDKGKSFTSD	0.5454	Non-allergen	Outside	Selected	
	WYPIEETCYYPKHIQYNILLGEGPC	0.7702	Non-allergen	Inside	Not-
Selected	
	RGDFNLLGIHDNCAVLPTHAECGDTILIDNIQ	0.5089	Non-allergen	Outside	Selected	
	PSKQSDSWLKKFTEWCNAAKGLEWIGYKI
SKFIDWLKEKL	0.3842	Allergen	Inside	Not-
Selected	
	TAAKHYAAQLFPLDINPEPITMEQAVYGTDYL
EPL	 0.8262	Non-allergen	Inside	Not-
Selected	
	FPGEKEPAALHDKDPRL	0.8541	Non-allergen	Outside	Selected	
	EKNLEYTQYCLNCLGSL	1.1994	allergen	Inside	Not-
Selected	
	DLNTSAGYPYVTLGIKK	1.0973	Non-allergen	Outside	Selected	
	PTVQRKKDTLLECKKLTLY	0.8374	Non-allergen	Inside	Not-
Selected	
	SLCLLAWHNGKQQYEDF	0.7100 	Non-allergen	Outside	Selected	
VP2	MTNPALMSPSIEACGFSDRLKQITIGSSTITTQ
DTLNT	0.5589	Non-allergen	Outside	Selected	
	NAQIRKKLELFTYVRFDLE	0.7074	Non-allergen	Inside	Not-
Selected	
	VPMAALEDKGKSFTSD	0.5454	Non-allergen	Outside	Selected	
	WYPIEETCYYPKHIQYNILLGEGPC	0.7702	Non-allergen	Inside	Not-
Selected	
	PTVQRKKDTLLECKKLTLY	0.8374	Non-allergen	Inside	Not-
Selected	
	TAGGENHVAFTDLRPY	1.0942	Non-allergen	Outside	Selected	
	TAAKHYAAQLFPLDINPEPITMEQAVYGTDYLEPL	0.8262	Non-allergen	Inside	Not-
Selected	
	ANVGNIVAIHVGGNGRVGYGAA	0.9524	Allergen	Inside	Not-
Selected	
	PKHFDGYCQQQVVMMDDLGQ	0.2462	Allergen	Inside	Not-
Selected	
	RGDFNLLGIHDNCAVLPTHAECGDTILIDNIQ	0.5089	Non-allergen	Outside	Selected	
	GKSLATTVIARGLTDSGNVYSLPP	0.1624	Allergen	Inside	Not-
Selected	
	LPITISIAPDKSEFSGARQSNK	0.8754	Allergen	Inside	Not-
Selected	
	DLNTSAGYPYVTLGIKK	1.0973	Non-allergen	Outside	Selected	


2C
	TAGGENHVAFTDLRPY	1.0942	Non-allergen	Outside	Selected	
	DLNTSAGYPYVTLGIKK	1.0973	Non-allergen	Outside	Selected	
	RGDFNLLGIHDNCAVLPTHAECGDTILIDNIQ	0.5089	Non-allergen	Outside	Not-
Selected	
	MTNPALMSPSIEACGFSDRLKQITIGSSTITTQDTLNT	0.5589	Non-allergen	Outside	Not-
Selected	
	NAQIRKKLELFTYVRFDLE	0.7074	Non-allergen	Inside	Not-
Selected	
	WYPIEETCYYPKHIQYNILLGEGPC	0.7702	Non-allergen	Inside	Not-
Selected	
	SLCLLAWHNGKQQYEDF	0.7100	Non-allergen	Outside	Selected	
	VRIISALTIVIRNSSD	0.4692 	Non-allergen	Inside	Not-
Selected	
	PTVQRKKDTLLECKKLTLY	0.8374	Non-allergen	Inside	Not-
Selected	
	RGDFNLLGIHDNCAVLPTHAECGDTILIDNIQ	0.5089	Non-allergen	Inside	Not-
Selected	
	KTKLYPSVFYDIFPGEKEPAALHDKDPRL	0.7670	Non-allergen	Outside	Not-
Selected	
	TAAKHYAAQLFPLDINPEPITMEQAVYGTDYLEPL	0.8262	Non-allergen	Inside	Not-
Selected	


Table 3. MHC-I and MHC-II epitope of Protein VP1
Type	Allele	Start	End	Peptide	Allergenicity	Antigenicity	Tropology	
MHC-I	HLA-A*02:03	9	17	ALHDKDPRL  	NA	1.3654 	outside 	
1.		HLA-B*15:01	5	13	ALEDKGKSF 	NA	1.0795	outside 	
1.		HLA-B*44:03	3	11	GEKEPAALH 	NA	0.8568	outside 	
1.		HLA-A*68:02	12	20	 EACGFSDRL 	NA	 0.7143	outside 	
1.		HLA-C*03:02	2	10	 AGGENHVAF 	NA	 0.4879	outside 	
1.		HLA-A*68:02	1	9	MTNPALMSP 	NA	0.4703	outside 	
1.		HLA-A*30:02	2	10	LNTSAGYPY 	NA	0.6086	outside 	
1.		HLA-A*30:02	2	10	 PGEKEPAAL 	NA	0.65	outside 	
1.		HLA-A*30:01	6	14	AGYPYVTLG 	NA	1.1	outside 	
1.		HLA-B*35:01	13	21	CAVLPTHAE 	NA	0.67	outside 	
1.		HLA-A*02:01	5	13	NLLGIHDNC 	NA	0.4638	outside 	
1.		HLA-A*02:01	6	14	LLGIHDNCA 	NA	0.5136	outside 	
1.		HLA-C*01:02	15	23	 VLPTHAECG 	NA	 0.5418 	outside 	
1.		HLA-B*40:01	2	10	GDFNLLGIH 	NA	1.3551	outside 	
1.		HLA-C*04:01	9	17	IHDNCAVLP 	NA	0.5853	outside 	
1.		HLA-A*68:02	1	9	 DLNTSAGYP 	NA	 0.7451	outside 	
1.		HLA-A*68:02	2	10	TNPALMSPS 	NA	0.5839	outside 	
1.		HLA-B*44:03	4	12	EKEPAALHD 	NA	0.7656	outside 	
1.		HLA-B*40:01	10	18	HDNCAVLPT 	NA	0.6714	outside 	
1.		HLA-B*51:01	4	12	PALMSPSIE 	NA	0.5263	outside 	
1.		HLA-C*08:02	7	15	PAALHDKDP 	NA	1.4635 	outside 	
1.		HLA-A*32:01	20	28	LKQITIGSS	NA	1.2232	inside	
1.		HLA-C*03:03	29	37	TITTQDTLN	NA	0.8494	inside	
1.		HLA-C*03:03	7	15	EDKGKSFTS	NA	0.3294	inside	
1.		HLA-C*06:02	17	25	PTHAECGDT	Allergen	0.5077	outside	
1.		HLA-A*24:02	7	15	PAALHDKDP	NA	1.4635	outside	
	HLA-C*07:01	1	9	SLCLLAWHN	Allergen	0.5692	outside	
1.		HLA-C*04:01	16	24	LPTHAECGD	NA	0.2105	outside	
1.		HLA-B*08:01	29	37	TITTQDTLN	NA	0.8494	inside	
1.		HLA-A*24:02	2	10	PMAALEDKG	Allergen	0.9914	outside	
1.		HLA-B*57:01	4	12	EKEPAALHD	NA	0.7656	outside	
1.		HLA-C*03:03	3	11	CLLAWHNGK	NA	0.1532	inside	
1.		HLA-A*68:02	3	11	CLLAWHNGK	NA	0.1532	inside	
1.		HLA-A*23:01	6	14	LLGIHDNCA	NA	0.5136	outside	
1.		HLA-A*01:01	7	15	WHNGKQQYE	NA	0.3478	inside	
1.		HLA-A*24:02	13	21	CAVLPTHAE	NA	0.6700	outside	
1.		HLA-B*07:02	2	10	GDFNLLGIH	NA	1.3551	outside	
MHC-II	HLADQA1*05:01/
DQB1*03:01	1	15	DLNTSAGYPYVTLGI 	NA	1.0886 	outside	
	HLA-DRB1*15:01	1	15	SLCLLAWHNGKQQYE   	NA	0.5412	outside	
	HLA-DQA1*05:01/
DQB1*03:01	2	16	LNTSAGYPYVTLGIK	NA	 1.3439	outside	
	HLA-DRB3*01:01	1	15	VPMAALEDKGKSFTS	NA	0.6721	outside	
	HLA-DRB1*09:01	2	16	LCLLAWHNGKQQYED 	NA	 0.6081	outside	
	HLA-DQA1*01:02/
DQB1*06:02	9	23	IHDNCAVLPTHAECG 	NA	0.5347	outside	
	HLA-DRB1*03:01	3	17	GEKEPAALHDKDPRL	NA	1.1311	outside	
	HLA-DPA1*02:01/
DPB1*14:01	2	16	PGEKEPAALHDKDPR	NA	0.9863	outside	
	HLA-DRB1*04:05	10	24	HDNCAVLPTHAECGD 	NA	0.5641	outside	
	HLA-DQA1*01:02/
DQB1*06:02	8	22	GIHDNCAVLPTHAEC 	NA	0.4831	outside	
	HLA-DRB1*13:02	11	25	IEACGFSDRLKQITI	NA	0.473	outside	
	HLA-DQA1*05:01/
DQB1*03:01	1	15	DLNTSAGYPYVTLGI  
	NA	1.0886	outside	
	HLA-DPA1*02:01/
DPB1*01:01	14	28	AVLPTHAECGDTILI
	Allergen	0.1986	outside	
	HLA-DRB1*08:02	15	29	VLPTHAECGDTILID	Allergen	0.0920	outside	
	HLA-DPA1*02:01/
DPB1*05:01	5	19	NLLGIHDNCAVLPTH
	Allergen	0.5683	outside	
	HLA-DRB5*01:01	15	29	VLPTHAECGDTILID	Allergen	 0.0920 	outside	
	HLA-DRB1*08:02	16	30	LPTHAECGDTILIDN	Allergen	-0.1547	outside	
	HLA-DPA1*01:03/
DPB1*04:01	23	37	ITIGSSTITTQDTLN
	Allergen	0.9862	inside	
	HLA-DQA1*01:01/
DQB1*05:01	12	26	NCAVLPTHAECGDTI
	NA	0.3588	outside	
	HLA-DQA1*01:01/
DQB1*05:01	8	22	GIHDNCAVLPTHAEC
	NA	0.4831	outside	
	HLA-DRB1*04:01	5	19	ALMSPSIEACGFSDR	Allergen	0.3667	outside	
	HLA-DRB1*07:01	18	32	THAECGDTILIDNIQ	Allergen	0.1958	outside	
	HLA-DPA1*03:01/
DPB1*04:02	10	24	SIEACGFSDRLKQIT
	NA	0.3611	inside	
	HLA-DPA1*02:01/
DPB1*05:01	5	19	ALMSPSIEACGFSDR
	Allergen	0.3667	outside	
	HLA-DQA1*05:01/
DQB1*03:01	1	15	DLNTSAGYPYVTLGI  
	NA	1.0886	outside	


Type	allele	start	end	peptide	Allergenicity	Antigenicity	Tropology	
MHC-I	HLA-A*68:02	12	20	EACGFSDRL 	NA	0.7143	outside	
	HLA-C*02:02	5	13	ALEDKGKSF 	NA	1.0795	outside	
	HLA-C*03:02	2	10	AGGENHVAF  	NA	0.4879	outside	
	HLA-A*68:02	1	9	MTNPALMSP 	NA	0.4703	outside	
	HLA-C*07:02	5	13	ALEDKGKSF 	NA	1.0795	outside	
	HLA-B*53:01	12	20	EACGFSDRL 	NA	0.7143	outside	
	HLA-A*30:02	2	10	LNTSAGYPY 	NA	0.6086	outside	
	HLA-A*01:01	2	10	LNTSAGYPY 	NA	0.6086	outside	
	HLA-A*26:01	5	13	ALEDKGKSF 	NA	1.0795	outside	
	HLA-A*02:03	5	13	NLLGIHDNC 	NA	0.4638	outside	
	HLA-C*12:02	13	21	CAVLPTHAE  	NA	0.6700	outside	
	HLA-A*68:02	5	13	ALEDKGKSF 	NA	1.0795	outside	
	HLA-C*05:01	15	23	VLPTHAECG 	NA	0.5418	outside	
	HLA-A*02:03	2	10	TNPALMSPS  	NA	0.5839	outside	
	HLA-B*15:01	1	9	DLNTSAGYP  	NA	0.7451	outside	
	HLA-C*06:02	2	10	TNPALMSPS  	NA	0.5839	outside	
	HLA-A*02:06	2	10	LNTSAGYPY 	NA	0.6086	outside	
	HLA-C*05:01	2	10	TNPALMSPS  	NA	0.5839	outside	
	HLA-C*07:04	10	18	HDNCAVLPT 	NA	0.6714	outside	
	HLA-A*24:02	1	9	DLNTSAGYP  	NA	0.7451	outside	
	HLA-A*02:03	2	10	GDFNLLGIH  	NA	1.3551	outside	
	HLA-C*05:01	2	10	GDFNLLGIH  	NA	1.3551	outside	
	HLA-C*02:09	10	18	HDNCAVLPT 	NA	0.6714	outside	
	HLA-C*02:02	10	18	HDNCAVLPT 	NA	0.6714	outside	
	HLA-C*07:02	4	12	PALMSPSIE 	NA	0.5263	outside	
	HLA-C*12:02	10	18	HDNCAVLPT 	NA	0.6714	outside	
	HLA-C*07:04	2	10	GDFNLLGIH  	NA	1.3551	outside	
	HLA-A*32:01	4	12	PALMSPSIE 	NA	0.5263	outside	
	HLA-A*32:01	2	10	TNPALMSPS  	NA	0.5839	outside	
	HLA-A*11:01	15	23	VLPTHAECG 	NA	0.5418	outside	
	HLA-C*01:02	2	10	GDFNLLGIH  	NA	1.3551	outside	
	HLA-B*40:01	4	12	PALMSPSIE 	NA	0.5263	outside	
	HLA-C*03:04	20	28	LKQITIGSS	NA	1.2232	inside	
	HLA-C*03:04	8	16	DKGKSFTSD	NA	-0.0082	outside	
	HLA-B*15:01	16	24	LPTHAECGD	NA	0.2105	outside	
	HLA-C*07:01	21	29	ECGDTILID	NA	-0.5371	inside	
	HLA-B*08:01	22	30	CGDTILIDN	NA	-0.9043 	inside	
	HLA-B*58:01	17	25	PTHAECGDT	allergen	0.5077	outside	
	HLA-B*35:01	9	17	PSIEACGFS	allergen	-0.6253	outside	
	HLA-B*44:02	29	37	TITTQDTLN	NA	0.8494	inside	
	HLA-A*31:01	4	12	FNLLGIHDN	Allergen	1.1654	outside	
	HLA-C*03:02	16	24	LPTHAECGD	NA	0.2105	outside	
	HLA-A*32:01	6	14	LEDKGKSFT	Allergen	0.9622 	inside	
	HLA-B*08:01	11	19	IEACGFSDR	Allergen	 0.7982	inside	
	HLA-A*33:01	27	35	SSTITTQDT	Allergen	0.9822	inside	
MHC-II	HLA-DQA1*05:01/
DQB1*03:01
	1	15	DLNTSAGYPYVTLGI 
	NA	1.0886	outside
	
	HLA-DQA1*05:01/
DQB1*03:01
	2	16	LNTSAGYPYVTLGIK 
	NA	1.3439	outside
	
Table 4. MHC-I and MHC-II epitope of Protein VP2

	HLA-DRB3*01:01	1	15	VPMAALEDKGKSFTS 	NA	0.6721	outside	
	HLA-DRB5*01:01	1	15	VPMAALEDKGKSFTS 	NA	0.6721	outside	
	HLA-DQA1*05:01/DQB1*02:01	1	15	DLNTSAGYPYVTLGI 	NA	1.0886	outside	
	HLA-DRB1*15:01	2	16	LNTSAGYPYVTLGIK 	NA	1.3439	outside	
	HLA-DQA1*01:02/DQB1*06:02	9	23	IHDNCAVLPTHAECG  	NA	0.5347	outside	
	HLA-DPA1*01:03/DPB1*02:01	2	16	LNTSAGYPYVTLGIK 	NA	1.3439	outside	
	HLA-DRB1*04:05			HDNCAVLPTHAECGD 	NA	0.5641	outside	
	HLA-DQA1*01:02/DQB1*06:02	8	22	GIHDNCAVLPTHAEC 	NA	0.4831	outside	
	HLA-DQA1*01:02/DQB1*06:02	10	24	HDNCAVLPTHAECGD 	NA	0.5641	outside	
	HLA-DQA1*01:02/DQB1*06:02	1	15	DLNTSAGYPYVTLGI 	NA	1.0886	outside	
	HLA-DRB1*13:02	1	15	VPMAALEDKGKSFTS 	NA	0.6721	outside	
	HLA-DRB1*04:05	9	23	IHDNCAVLPTHAECG  	NA	0.5347	outside	
	HLA-DQA1*05:01/DQB1*02:01	2	16	LNTSAGYPYVTLGIK 	NA	1.3439	outside	
	HLA-DRB1*13:02	11	25	IEACGFSDRLKQITI  	NA	0.4730	outside	
	HLA-DRB4*01:01	1	15	VPMAALEDKGKSFTS 	NA	0.6721	outside	
	HLA-DRB1*12:01	1	15	VPMAALEDKGKSFTS 	NA	0.6721	outside	
	HLA-DRB1*12:01	1	15	DLNTSAGYPYVTLGI 	NA	1.0886	outside	
	HLA-DRB1*04:01	10	24	HDNCAVLPTHAECGD 	NA	0.5641	outside	
	HLA-DRB1*07:01	2	16	LNTSAGYPYVTLGIK 	NA	1.3439	outside	
	HLA-DRB1*12:01	10	24	HDNCAVLPTHAECGD 	NA	0.5641	outside	
	HLA-DRB1*09:01	1	15	DLNTSAGYPYVTLGI 	NA	1.0886	outside	
	HLA-DRB1*11:01	11	25	IEACGFSDRLKQITI  	NA	0.4730	outside	
	HLA-DRB1*15:01	1	15	DLNTSAGYPYVTLGI 	NA	1.0886	Outside	
	HLA-DRB3*02:02	8	22	GIHDNCAVLPTHAEC 	NA	0.4831	Outside	
	HLA-DRB1*07:01	10	24	HDNCAVLPTHAECGD 	NA	0.5641	Outside	
	HLA-DRB1*04:05	8	22	GIHDNCAVLPTHAEC 	NA	0.4831	Outside	
	HLA-DQA1*03:01/DQB1*03:02	1	15	DLNTSAGYPYVTLGI 	NA	1.0886	Outside	
	HLA-DQA1*01:01/DQB1*05:01	2	16	LNTSAGYPYVTLGIK 	NA	1.3439	Outside	
	HLA-DQA1*05:01/DQB1*03:01	8	22	GIHDNCAVLPTHAEC 	NA	0.4831	outside	
	HLA-DPA1*01:03/DPB1*04:01	2	16	LNTSAGYPYVTLGIK 	NA	1.3439	Outside	
	HLA-DRB1*08:02	11	25	IEACGFSDRLKQITI  	NA	0.4730	outside	
	HLA-DRB3*01:01	11	25	IEACGFSDRLKQITI  	NA	0.4730	outside	
	HLA-DRB1*04:01	9	23	IHDNCAVLPTHAECG  	NA	0.5347	outside	
	HLA-DRB1*04:01	1	15	VPMAALEDKGKSFTS 	NA	0.6721	outside	
	HLA-DPA1*02:01/DPB1*05:01	2	16	LNTSAGYPYVTLGIK 	NA	1.3439	outside	
	HLA-DRB1*12:01	9	23	IHDNCAVLPTHAECG  	NA	0.5347	outside	
	HLA-DQA1*01:02/DQB1*06:02	2	16	LNTSAGYPYVTLGIK 	NA	1.3439	outside	
	HLA-DPA1*03:01/DPB1*04:02	13	27	CAVLPTHAECGDTIL	NA	0.2742	outside	
	HLA-DRB1*15:01	15	29	VLPTHAECGDTILID	Allergen	0.0920	outside	
	HLA-DRB1*04:05	13	27	ACGFSDRLKQITIGS	NA	1.1662	inside	
	HLA-DRB1*04:05	9	23	PSIEACGFSDRLKQI	NA	0.2807	outside	
	HLA-DQA1*01:01/DQB1*05:01	14	28	AVLPTHAECGDTILI	Allergen	0.1986	outside	
	HLA-DRB1*13:02	3	17	NPALMSPSIEACGFS	NA	-0.0279	outside	
	HLA-DPA1*03:01/DPB1*04:02	18	32	THAECGDTILIDNIQ	Allergen	0.1958	inside	
	HLA-DRB1*07:01	4	18	PALMSPSIEACGFSD	Allergen	-0.0673	outside	
	HLA-DRB1*04:05	13	27	ACGFSDRLKQITIGS	NA	1.1662	inside	


Table 5. MHC-I and MHC-II epitope of Protein 2C

Type	Allele	Start	End	Peptide	Antigenicity	Allergenicity	Tropology	
MHC-I	HLA-C*03:02	2	10	AGGENHVAF	0.4879	NA	outside	
	HLA-A*30:02	2	10	LNTSAGYPY	0.6086	NA	outside	
	HLA-A*68:02	1	9	DLNTSAGYP	0.7451	NA	outside	
	HLA-A*02:03	8	16	HNGKQQYED	0.7604	NA	Inside	
	HLA-A*26:01	2	10	LCLLAWHNG	0.1981	Allergen	outside	
	HLA-A*24:02	4	12	GENHVAFTD	0.9339	Allergen	outside	
	HLA-B*44:02	8	16	HNGKQQYED	0.7604	NA	inside	
	HLA-B*07:02	3	11	GGENHVAFT	0.7083	Allergen	outside	
	HLA-A*24:02	1	9	SLCLLAWHN	0.5692	Allergen	outside	
	HLA-B*07:02	3	11	CLLAWHNGK	0.1532	NA	inside	
	HLA-A*26:01	7	15	WHNGKQQYE	0.3478	NA	inside	
	HLA-B*35:01	4	12	LLAWHNGKQ	0.1498	Allergen	inside	
	HLA-A*30:01	8	16	HNGKQQYED	0.7604	NA	inside	
	HLA-A*26:01	3	11	CLLAWHNGK	0.1532	NA	inside	
MHC-II	HLA-DQA1*05:01/
DQB1*03:01	1	15	DLNTSAGYPYVTLGI 	1.0886	NA	outside	
	HLA-DRB1*15:01	1	15	SLCLLAWHNGKQQYE  	0.5412	NA	outside	
	HLA-DQA1*05:01/
DQB1*03:01	2	16	LNTSAGYPYVTLGIK 	1.3439	NA	outside	
	HLA-DRB1*09:01	2	16	LCLLAWHNGKQQYED 	0.6081	NA	outside	
	HLA-DRB1*11:01	1	15	DLNTSAGYPYVTLGI	1.0886	NA	outside	
	HLA-DPA1*02:01/
DPB1*14:01	1	15	SLCLLAWHNGKQQYE	0.4621	NA	outside	
	HLA-DPA1*03:01/
DPB1*04:02	3	17	CLLAWHNGKQQYEDF	0.6378	NA	inside	
	HLA-DPA1*02:01/
DPB1*01:01	3	17	CLLAWHNGKQQYEDF	0.6378	NA	inside	
	HLA-DPA1*03:01/
DPB1*04:02	2	16	LCLLAWHNGKQQ YED	0.6081	NA	outside	
	HLA-DRB1*13:02	1	15	TAGGENHVAFTDLRP	1.2949	Allergen	outside	
	HLA-DPA1*03:01/
DPB1*04:02	2	16	LNTSAGYPYVTLGIK	1.3439	NA	outside	
	HLA-DRB1*08:02	3	17	NTSAGYPYVTLGIKK	1.2110	Allergen	outside	
	HLA-DRB1*11:01	1	15	TAGGENHVAFTDLRP	1.2949	Allergen	outside	
	HLA-DPA1*01:03/
DPB1*02:01	1	15	SLCLLAWHNGKQQYE	0.5412	NA	outside	


Table 6. List of the predicted B-cell epitopes 


Type	Peptide	Allergenicity	Antigenicity	Tropology	
VP1	LMSPSIEACGFSDRLKQITIG
SSTITTQ	0.5643 	Non-allergen	Outside	
	KEPAALHDKD	1.2349 	Non-allergen	Outside	
	YPYVTL	1.0891	Non-allergen	Outside	
	LEDKGKSF	1.2675	Allergen	Outside	
	HAECGDTI	0.5821	Allergen	Inside	
	VAFTD	0.4021	Allergen	Inside	
	EPAALHDKDPRLDLNT	0.8258 	Non-allergen	Outside	
VP2	QDLNTSAGYPYVTLGI	1.0594	Non-allergen	Outside	
	IEACGFS	-0.8281	Allergen	Outside	
	GYPYV	-	Allergen	Outside	
	CGFSDRLKQITIGSST	1.1049	Non-allergen	Inside	
	NCAVLPTH	0.6689	Allergen	Inside	
	TSDRGDFNLLGIHDNC	0.8370	Allergen	Outside	
2C	VAFTD	-	Allergen	Inside	
	GYPYV	-	Allergen	Outside	
	YPYVTL	1.0891	Non-allergen	Outside	
	KSLCLLAWHNGKQQYE	0.7052	Non-allergen	Outside	
	ENHVAFTDLRPYDLNT	1.4419	Non-allergen	Inside	


Table 7. Proportion of the population coverage of vaccine


Region	Population coverage	Average number of epitope hits	minimum number of epitope hits	
East Asia	56.97%	1.09	0.23	
Northeast Asia	75.84%	1.8	0.41	
South Asia	59.62%	1.26	0.25	
Southeast Asia	75.83%	1.75	0.41	
Southwest Asia	63.79%	1.4	0.28	
Europe	67.95%	1.53	0.31	
East Africa	89.14%	2.45	0.92	
West Africa	92.56%	2.66	1.49	
Central Africa	85.49%	2.2	0.69	
North Africa	74.84%	1.77	0.4	
South Africa	34.43%	0.38	0.15	
West Indies	72.39%	1.69	0.36	
North America	79.4%	1.88	0.49	
Central America	72.67%	1.47	0.37	
South America	69.9%	1.55	0.33	
Oceania	74.15%	1.75	0.39	
World	68.6%	1.54	0.32	


Table 8. Protein Disulfide engineering Scores by DbD2 server in terms of Energy value


Res1 Chain	Res1 Seq 	Res1 AA	Res2 Chain	Res2 Seq	Res2 AA	Chi3	Energy	Sum B-Factors	
A	65	PHE	A	69	LEU	121.35	5.61	199.98	
A	163	GLU	A	171	ALA	109.19	3.69	199.98	
A	168	ALA	A	171	ALA	-60.78	6.49	199.98	
A	229	GLY	A	236	ILE	111.64	3.56	199.98	
A	235	GLY	A	394	HIS	-97.47	2.55	199.98	
A	279	GLY	A	283	THR	116.96	2.58	199.98	
A	306	PRO	A	391	LEU	83.41	4.29	199.98	
A	340	GLY	A	421	THR	-91.12	4.4	199.98	
A	360	GLU	A	363	GLY	71.01	4.95	199.98	
A	361	CYS	A	373	CYS	-63.07	3.42	199.98	
A	371	ASP	A	374	ALA	83.04	1.43	199.98	
A	379	HIS	A	396	GLY	116.23	2.67	199.98	
A	416	LYS	A	454	VAL	-76.99	4.42	199.98	
A	420	VAL	A	423	LYS	-101.77	1.53	199.98	


Table 9. Predicted Linear epitopes from V2 vaccine protein using Ellipro server


No.	Chain	Start	End	Peptides	No. of residues	Scores	
1	A	746	444	NLLKTINKSALETKTTTKLSMLELHGNPFECTCDIGD
FRRWMDEHLNVKIPRLVDVICASPGDQRGKSIVSL
EEAAAKMAENPNIDDLPAPLL
AALGAADLALNELVERGEAALQPRLDLNTKKQD	126	0.839	
2	A	516	533	SNAQVLSGTEFSAIPHVK	18	0.804	
3	A	491	510	NSIFFIGPNQFENLPDIACL	20	0.774	
4	A	540	558	NRLDFDNASALTELSDLEV	19	0.729	
5	A	565	587	SHYFRIAGVTHHLEFIQNFTNLK	23	0.712	
6	A	596	610	IYTLTDKYNLESKSL	15	0.696	
7	A	97	127	NPNVGLNITDGAFLNLKNL	19	0.67	
8	A	722	738	NRISHLPSGFLSEVSSL	17	0.659	
9	A	621	641	DILWNDDDNRYISIFKGLKNL	21	0.644	
10	A	480	486	AAYGKAL	7	0.642	
11	A	40	95	KQNDSVIAECSNRRLQEVPQTVGKYVTELDLSDN
FITHITNESFQGLQNLTKINLN	56	0.64	
12	A	129	171	ELLLEDNQLPQIPSGLPESLTELSLIQNNIYNITKE
GISRLIN	43	0.64	
13	A	700	713	LLFLTDSLSDFTSS	14	0.597	
14	A	651	662	LKHIPNEAFLNL	12	0.54	
15	A	184	187	NKVC	4	0.516	
16	A	676	687	LKFFNWTLLQQF	12	0.514	


Table 10. Discontinuous B-cell epitopes from V2 vaccine protein using Ellipro server
No.	Residues	No. of residues	Scores	
1	A:E1, A:A2, A:A3, A:A4, A:K5, A:M6, A:A7, A:E8, A:N9, A:P10, A:N11, A:I12, A:D13, A:D14, A:L15, A:P16, A:A17, A:P18, A:L19, A:L20, A:A21, A:A22, A:L23, A:G24, A:A25, A:A26, A:D27, A:L28, A:A29, A:L30	30	0.981	
2	A:G107, A:E108, A:A109, A:A110, A:L111, A:Q112	6	0.958	
3	A:N101, A:E102, A:L103, A:V104, A:E105, A:R106	6	0.953	
4	A:R338, A:E340, A:S418, A:L420, A:E421, A:I422, A:I423, A:L425, A:I430, A:S431, A:P432, A:L433, A:N466, A:H469, A:F470, A:T471, A:R472, A:P473, A:L474, A:I475, A:K476, A:P477, A:Q478, A:C479, A:A480, A:A481, A:Y482, A:G483, A:K484, A:A485, A:L486, A:L488, A:N491, A:S492, A:I493, A:F494, A:F495, A:I496, A:G497, A:P498, A:N499, A:Q500, A:F501, A:E502, A:N503, A:L504, A:P505, A:D506, A:I507, A:A508, A:C509, A:L510, A:L512, A:N515, A:S516, A:N517, A:A518, A:Q519, A:V520, A:L521, A:S522, A:G523, A:T524, A:E525, A:F526, A:S527, A:A528, A:I529, A:P530, A:H531, A:V532, A:K533, A:N540, A:R541, A:L542, A:D543, A:F544, A:D545, A:N546, A:A547, A:S548, A:A549, A:L550, A:T551, A:E552, A:L553, A:S554, A:D555, A:L556, A:E557, A:V558, A:N564, A:S565, A:H566, A:Y567, A:F568, A:R569, A:I570, A:A571, A:G572, A:V573, A:T574, A:H575, A:H576, A:L577, A:E578, A:F579, A:I580, A:Q581, A:N582, A:F583, A:T584, A:N585, A:L586, A:K587, A:N594, A:N595, A:I596, A:Y597, A:T598, A:L599, A:T600, A:D601, A:K602, A:Y603, A:N604, A:L605, A:E606, A:S607, A:K608, A:S609, A:L610, A:V611, A:R619, A:D621, A:I622, A:L623, A:W624, A:N625, A:D626, A:D627, A:D628, A:N629, A:R630, A:Y631, A:I632, A:S633, A:I634, A:F635, A:K636, A:G637, A:L638, A:K639, A:N640, A:L641, A:R650, A:L651, A:K652, A:H653, A:I654, A:P655, A:N656, A:E657, A:A658, A:L660, A:N661, A:L662, A:M675, A:F679, A:N680, A:T682, A:L683, A:Q685, A:Q686, A:F687, A:K699, A:L700, A:L701, A:F702, A:L703, A:T704, A:D705, A:S706, A:L707, A:S708, A:D709, A:F710, A:T711, A:S712, A:S713, A:L714, A:R715, A:N722, A:R723, A:I724, A:S725, A:H726, A:L727, A:P728, A:S729, A:G730, A:F731, A:L732, A:S733, A:E734, A:V735, A:S736, A:S737, A:L738, A:K739, A:L743, A:S745, A:N746, A:L747, A:L748, A:K749, A:T750, A:I751, A:N752, A:K753, A:S754, A:A755, A:L756, A:E757, A:T758, A:K759, A:T760, A:T761, A:T762, A:K763, A:L764, A:S765, A:L767, A:L769, A:H770, A:G771, A:N772, A:P773, A:F774, A:E775, A:C776, A:T777, A:C778, A:D779, A:I780, A:G781, A:D782, A:F783, A:R784, A:R785, A:W786, A:M787, A:D788, A:E789, A:H790, A:L791, A:N792, A:V793, A:K794, A:I795, A:P796, A:R797, A:L798, A:V799, A:D800, A:V801, A:I802, A:C803, A:A804, A:S805, A:P806, A:G807, A:D808, A:Q809, A:R810, A:G811, A:K812, A:S813, A:I814, A:V815, A:S816, A:L817, A:E818	283	0.682	
5	A:N51, A:R52, A:L54, A:Q55, A:E56, A:V57, A:P58, A:Q59, A:T60, A:V61, A:K63, A:V65, A:T66, A:E67, A:L68, A:D69, A:L70, A:S71, A:D72, A:N73, A:F74, A:I75, A:T76, A:H77, A:I78, A:T79, A:N80, A:E81, A:S82, A:F83, A:Q84, A:L86, A:I92, A:N97, A:P98, A:N99, A:V100, A:L114, A:N115, A:I116, A:T117, A:D118, A:G119, A:A120, A:F121, A:L122, A:N123, A:L124, A:K125, A:N126, A:L127, A:L130, A:L131, A:L132, A:E133, A:N135, A:Q136, A:L137, A:P138, A:Q139, A:I140, A:P141, A:S142, A:G143, A:L144, A:P145, A:S147, A:L148, A:T149, A:E150, A:L151, A:S152, A:I154, A:Q155, A:N156, A:N157, A:I158, A:Y159, A:N160, A:T162, A:K163, A:E164, A:G165, A:S167, A:L169, A:L177, A:A178, A:C181, A:K185, A:V186, A:C187, A:E188, A:K189, A:T190, A:N191, A:G195, A:V196, A:S207, A:N211, A:G255, A:C257, A:P258, A:F261, A:P266, A:C267, A:V268, A:P269, A:C270, A:D271, A:G272, A:G273, A:S275	112	0.644	
6	A:P35, A:C36, A:Q41, A:N42, A:D43, A:V45, A:I46, A:A47, A:E48, A:S50, A:T90	11	0.643	
7	A:L436, A:D437, A:L438, A:N439, A:T440, A:K441, A:K442, A:Q443, A:D444	9	0.598	
8	A:N88, A:L89, A:I161, A:I166, A:I170, A:N171	6	0.518	
